# Targeted mutagenesis of *BnaSTM* leads to abnormal shoot apex development and cotyledon petiole fusion at the seedling stage in *Brassica napus* L.

**DOI:** 10.3389/fpls.2023.1042430

**Published:** 2023-02-14

**Authors:** Kaidi Yu, Huailin Li, Xiaolong Wu, Olalekan Amoo, Hanzi He, Chuchuan Fan, Yongming Zhou

**Affiliations:** ^1^ Hubei Hongshan Laboratory, Wuhan, Hubei, China; ^2^ National Key Laboratory of Crop Genetic Improvement, Huazhong Agricultural University, Wuhan, Hubei, China

**Keywords:** *Brassica napus* L., CRISPR/Cas9, shoot apical meristem (SAM), *BnaSTM*, fused cotyledon petiole

## Abstract

The Arabidopsis homeodomain transcription factor *SHOOT MERISTEMLESS* (*STM*) is crucial for shoot apical meristem (SAM) function, which cooperates with *CLAVATA3* (*CLV3*)*/WUSCHEL* (*WUS*) feedback regulation loops to maintain the homeostasis of stem cells in SAM. *STM* also interacts with the boundary genes to regulate the tissue boundary formation. However, there are still few studies on the function of *STM* in *Brassica napus*, an important oil crop. There are two homologs of *STM* in *B. napus* (*BnaA09g13310D* and *BnaC09g13580D*). In the present study, CRISPR/Cas9 technology was employed to create the stable site-directed single and double mutants of the *BnaSTM* genes in *B. napus.* The absence of SAM could be observed only in the *BnaSTM* double mutants at the mature embryo of seed, indicating that the redundant roles of *BnaA09.STM* and *BnaC09.STM* are vital for regulating SAM development. However, different from Arabidopsis, the SAM gradually recovered on the third day after seed germination in *Bnastm* double mutants, resulting in delayed true leaves development but normal late vegetative and reproductive growth in *B. napus*. The *Bnastm* double mutant displayed a fused cotyledon petiole phenotype at the seedling stage, which was similar but not identical to the *Atstm* in Arabidopsis. Further, transcriptome analysis showed that targeted mutation of *BnaSTM* caused significant changes for genes involved in the SAM boundary formation (*CUC2*, *CUC3*, *LBDs*). In addition, *Bnastm* also caused significant changes of a sets of genes related to organogenesis. Our findings reveal that the *BnaSTM* plays an important yet distinct role during SAM maintenance as compared to Arabidopsis.

## 1 Introduction

Rapeseed (*Brassica napus* L., AACC, 2n = 38) is the third-largest oilseed crop worldwide after soy bean and oil palm, accounting for about 13% of the global total production of vegetable oil (USDA ERS, 2021). Shoot apical meristem (SAM) is a crucial structure for forming the aerial organs of plants. Inside it, the dynamic balance between stem cell division and differentiation is required to maintain plant normal growth and development. The cooperation of SAM development will facilitate systematic design for high yield breeds in agriculture (Xue et al., 2020). For example, in *B. napus*, the development of SAM can affect the plant architecture to generate multi-inflorescence structure (Lu et al., 2022). In addition, by editing the important genes in SAM, we can obtain multilocular silique, which is also a desired trait for the development of high-yield varieties of Brassica (Yang et al., 2018). Therefore, it is of great significance to study the development of SAM for the breed improvement of rapeseed.

The *KNOTTED-LIKE HOMEOBOX* (*KNOX*) transcription factors are important regulators for the formation of SAM, which contribute to plant growth and development in all stages (Scofield and Murray, 2006; Barton, 2010; Roth et al., 2018). There are four Class I KNOX genes, i.e., *KNOTTED-LIKE FROM ARABIDOPSIS THALIANA 1* (*KNAT1*)/*BREVIPEDICELLUS* (*BP*), *KNAT2*, *KNAT6* and *SHOOT MERISTEMLESS* (*STM*). The expression of *KNAT1* is limited to the subcutaneous cells of the stem and pedicel, which affects the intermodal development to regulate the plant height (Venglat et al., 2002). *KNAT2* and *KNAT6* are involved in regulating inflorescence development (Ragni et al., 2008). *KNAT6* has also been confirmed to be involved in the formation of organ boundaries during embryogenesis (Belles-Boix et al., 2006). Several research studies revealed that *STM* functions during embryonic and postembryonic development in the formation and maintenance of SAM (Hay and Tsiantis, 2010). Cell-to-cell communication in plants includes the selective trafficking of transcription factors and other signals through plasmodesmata. The *KNOX I* family transcription factors, which use this pathway, are essential for stem cell establishment and/or maintenance. *CHAPERONIN CONTAINING TCP1 SUBUNIT 8* (*CCT8*) is a subunit of the chaperonin complex required for gene transport in the *KNOX I* family, which maintains stem cell homeostasis by affecting the intercellular transport of *STM* proteins (Xu et al., 2011).

*CLAVATA3* (*CLV3*)/*WUSCHEL* (*WUS*) feedback regulation loop is the key pathway regulating the proliferation and differentiation of stem cells in SAM (Schoof et al., 2000). Previous studies have reported that *STM* uses a different approach from *WUS* to inhibit the cell differentiation maintaining the function of SAM in Arabidopsis (Brand et al., 2002; Gallois et al., 2002; Lenhard et al., 2002; Cole and Nolte C Werr, 2006). Recent reports found that *STM* is expressed in the whole meristematic tissue and forms a heterodimer with *WUS* through direct interaction. The heterodimers bind at the *CLV3* promoter site to promote *CLV3* expression, confining the *WUS*-expressing cell population within the organizing center (Su et al., 2020).

Moreover, the interaction between *STM* and plant hormones is considered a balancing mechanism outside the *CLV3/WUS* pathway (Song et al., 2017). *STM* activates the transcription of the cytokinin (CK) synthetic gene *ISOPENTENYL TRANSFEREASE7* (*IPT7*) to increase the cytokinin level in the central zone cells of the SAM (Yanai et al., 2005), whereas it inhibits the gibberellin (GA) synthetic gene *GA 20-oxidase1* (*GA20ox1*) expressed at the base of SAM (Jasinski et al., 2005). Therefore, creating a “high CK and low GA” microenvironment in SAM, which is of great significance to maintaining the SAM functionality (Belles-Boix et al., 2006). *FT INTERACTING PROTEIN 3* (*FTIP3*) and *FTIP4* can promote the balance between the maintenance and differentiation of SAM by coordinating intracellular and intercellular transport of *STM* (Liu et al., 2018). There are also research findings showing that *STM* inhibits cellular differentiation and endoreduplication, acting through CK and the CK-inducible *CYCLIN-D3* (*CYCD3*) cell cycle regulators, establishing a mechanistic link to cell cycle control which provides sustained mitotic activity to maintain a pool of undifferentiated cells in the SAM (Scofield et al., 2013). *STM* is also associated with auxin and lateral organ activation-related genes such as *AXIAL REGULATOR YABBY 3* (*YAB3*), *ASYMMETRIC LEAVES 1* (*AS1*), *ASYMMETRIC LEAVES 2* (*AS2*), and *JAGGED LATERAL ORGAN* (*JLO*) (Kumaran et al., 2002; Guo et al., 2008; Bureau et al., 2010; Rast MI Simon, 2012).

In addition to regulating meristem identity, STM also functions in boundary specification and leaf shape. In Arabidopsis, the organ-boundary-associated genes *CUP-SHAPED COTYLEDON1* (*CUC1*), *CUC2*, and *CUC3* are required to activate *STM* expression and the subsequent formation of the SAM during embryogenesis (Vroemen et al., 2003). During embryonic development, *CUCs* and *STM* regulate the expression of each other. *CUC1* and *CUC2* trigger *STM* expression at the globular stage (Takada et al., 2001). During postembryonic development, *STM* promotes the expression of *CUC1/2/3* (Kwon et al., 2006; Boscá et al., 2011; Spinelli et al., 2011). Research has shown a direct positive transcriptional feedback loop between *STM* and *CUC1*, despite their distinct expression patterns in the meristem and organ boundary. Moreover, *STM* can activate the expression of the *CUC1*-targeting microRNA *miR164c* (Scofield et al., 2018). Moreover, in Arabidopsis, it has been reported that the complexity of the leaf shape is related to the expression of *STM* in the leaves (Piazza et al., 2010). Recent findings revealed that *REDUCED COMPLEXITY* (*RCO*) and *STM* were able to form a complex leaf shape similar to *Cardamine hirsuta* (Kierzkowski et al., 2019).

The association of SAM with yield traits in tomato, maize, rice, and rapeseed has been confirmed (Doebley, 2004; Elhiti et al., 2013; Ohmori et al., 2013; Fan et al., 2014; Perales et al., 2016). However, few reports are available on *STM*-related research in *B. napus* except that altered expression of *BnSTM* affects the morphology, behavior, and quality of microspore-derived embryos (MDEs) (Elhiti et al., 2013). The present study employed the CRISPR/Cas9 system to obtain the efficient knockout of *STM* homologs in *B. napus* through stable *Agrobacterium*-mediated transformation. Abnormal shoot apex development and cotyledon petiole fusion at the seedling stage was observed in the double mutants. Furthermore, the transcriptomics analysis of the *Bnastm* mutant was also used to investigate the molecular mechanism.

## 2 Materials and methods

### 2.1 Plant materials

The semi‐winter *B. napus* pure line J9707 was used as the transformation receptor in this study, and the seeds were obtained from the National Engineering Research Center of Rapeseed, Wuhan, China.

### 2.2 Cultivation conditions

T_0_ and T_1_ transgenic plants and WT plants grew in greenhouse (16/8 h of light/dark at 22°C). In the winter-type oilseed rape growing season, the homozygous mutant T_3_ lines without T-DNA were selected to grow in the experimental farm of Huazhong Agriculture University, Wuhan, China. The field experiment was conducted with a randomized complete block design and repeated three times. 11-12 plants are planted in each row, and the spacing between plants in each row is 21 cm, and the spacing between rows is 30 cm. The field management was performed in line with standard breeding practices.

### 2.3 Construction of the CRISPR/Cas9 vector and plant transformation

The CRISPR/Cas9 genome-editing system was utilized for gene editing of *BnaSTM* in this study. To construct the Cas9/sgRNA-expressing binary vectors, sequence-specific sgRNAs in the target gene were selected using the Web-based tool CRISPR-P2.0 (http://crispr.hzau.edu.cn/cgi-bin/CRISPR2). The binary pYLCRIPSR/Cas9 multiplex genome targeting vector system was provided by Prof. Yaoguang Liu (South China Agriculture University, Guangzhou, China) and used for construct assembly according to the method described by Ma et al. (2015). The oligos used in constructing the sgRNA vectors are listed in **Table S1**. The resulting construct contained a Cas9p expression cassette, sgRNA expression cassettes with target sequences and a hygromycin resistance cassette.

The *Agrobacterium tumefaciens*-mediated hypocotyl method (Zhou and Fowke 2002) was used to transform the resulting constructs into *B. napus* (Zhou et al., 2002). The transgenic plants were screened and confirmed by antibiotic selection and PCR.

### 2.4 Identification of transgenic plants and potential off-targets

The presence of the T-DNA in the construct was identified using the specific primer pairs PB-L/PB-R (**Table S1**) employing PCR.

The edit detection in transgenic plants using the high-throughput tracking of mutations (Hi-TOM) platform (Yang et al., 2017). The corresponding online tool (http://www.hi-tom.net/hi-tom/) was utilized to analyze the obtained sequencing data and track the mutation in the target sites. The target-specific primer sets are listed in **Table S1**. Furthermore, the putative off-target sites (7 annotated genes for sgRNA-1 and 10 for sgRNA-2) were identified using CRISPR-P2.0 (http://crispr.hzau.edu.cn/CRISPR2/) against the reference (*Brassica napus* v4.1).

### 2.5 Sequence analysis

Multiple sequence alignment of nucleotide and amino acid sequences was performed by ClustalW (http://www.clustal.org/). Motif and domain analysis of amino acid sequences were performed using MEME (https://meme-suite.org/meme/tools/meme), CDD (https://www.ncbi.nlm.nih.gov/Structure/bwrpsb/bwrpsb.cgi) and Pfam (http://pfam.xfam.org/). The homologous amino acid sequences of *STM* in different species were collected, and the phylogenetic analyses were constructed by MEGA11 software.

### 2.6 RNA extraction and qRT-PCR

The samples were collected from double mutants (STM-11-7-8-14, STM-14-1-11-9, STM-8-5-1-9), *BnaA09.STM* single mutants (STM-8-5-10-21, STM-14-8-12-5), *BnaC09.STM* single mutants (STM-7-7-9-2, STM-11-10-3-6) and WT (J9707). At least three independent extractions were conducted per sample. The sampling period is the 7th day after germination, The sampled tissue is the fused cotyledon petiole containing the SAM for RNA-seq sequencing. We extracted root (seedling stages), leaf (seedling stages), lower stem (bolting stages), bud (bolting stages), flower (flowering stages), SAM (seedling stages), and 7 DAF seeds from WT planted at the same time with the mutant of T_3_ generation as samples to detect gene expression in different tissues. Total RNA was extracted using the EasyPure Plant RNA Kit (TransGen Biotech, Beijing, China), and cDNA was synthesized using the Transcript RT Kit (TransGen Biotech). To perform the qPCR, the TransStart Top Green qPCR SuperMix Kit (TransGen Biotech) was used employing a CFX384 Real-Time System (Bio-Rad). The relative quantification was performed using the comparative cycle threshold method. The relative amount of PCR product which was amplified using the designed primer sets (**Table S1**), was normalized to the reference genes, *BnaACT7* and *BnaUBC9*. The data from three biological replicates were analyzed following the relative quantification method (2^−ΔΔCT^).

### 2.7 RNA-seq transcriptomic analysis

Samples collection of double mutants and WT, and RNA extraction as described in the above section. The process of cDNA library construction, sequencing, quality control, data filtering, and read mapping to the reference genome (*Brassica napus* v4.1), identification of differentially expressed genes (DEGs) using DESeq2 and GO. KEGG pathway enrichment analysis of DEGs was performed as described by Shahid et al. (2019). The fragments per kilobase of transcript per million mapped reads (FPKM) were calculated as a measure of gene expression level. The genes with a false discovery rate (FDR) ≤0.05 and an absolute value of log_2_(fold change) ≥ 1.5 between mutant and wild type (WT) were defined as DEGs. The raw sequence data were submitted in the NCBI Sequence Read Archive (PRJNA769039).

### 2.8 Paraffin section preparation

Seeds at different germination stages were collected from the double mutants (STM-11-7-8-14, STM-14-1-11-9, STM-8-5-1-9) and WT. Furthermore, immediately fixed for 24 h at 4°C in a fixation solution containing 5% acetic acid, 3% formaldehyde, and 50% ethanol. Embedding, sectioning, and staining with Calcium polystyrene sulphonate were performed as described by Chaplin et al (Chaplin, 1999). Images were obtained using a Nikon ECLIPSE 80i compound microscope.

### 2.9 Determination of protein and fatty acid content in seeds

The seeds collected from the WT, *BnaA09.STM* single mutants (aaCC) and double mutants (aacc) lines were used for fatty acid composition, protein and glucosinolate content analysis by near-infrared spectroscopy at the National Engineering Research Center of Rapeseed (Huazhong Agricultural University, Wuhan, China) and the near-infrared spectroscopy analysis model of each substance was constructed by the National Engineering Research Center of Rapeseed.

### 2.10 Statistical analysis

Statistical analyses were performed using the IBM SPSS Statistics program (version 25). Comparisons of distributions between 2 groups or between 3 groups were made by LSD (Least—Significant Difference) test or Student’s t test. Correlations between 2 variables were tested with Spearman’s rho correlation coefficients.

## 3 Results

### 3.1 Molecular cloning and characterization of *STM* homologs in *B napus*

Previous studies have shown that *STM* is a key regulator during SAM development in many plant species. A BLASTP search identified two close homologs of *STM* in the rapeseed genome (*BnaA09g13310D* and *BnaC09g13580D*) and these *STM* homologs were named *BnaA09*.*STM* and *BnaC09*.*STM*. DNA and cDNA sequences of *STM* homologs were cloned to confirm their gene structure as well as check the putative mutations in transformation receptor J9707 (**Figure S1**). The *BnaA09*.*STM* shared 94.87% similarity with *BnaC09*.*STM* at the amino acid level.

*AtSTM*, *BnaA09.STM* and *BnaC09.STM* contains four conserved domains in their amino acid sequences, namely, KNOX1, KNOX2, ELK, and HD, indicating that these genes encode proteins with similar functions (**Figure S2**). The motif analysis of *KNOX I* genes in different crops indicated that the homologous genes of *STM* in *Brassica* species had an additional motif at the front of protein sequence than Arabidopsis, which function was still unknown. This suggests the functional differentiation of *STM* during the evolution process in *Brassica* (**Figure S3**). The phylogenetic analyses revealed that all *STM* homologs from different *Brassica* species were clustered together with *AtSTM*, whereas the *STM* homologs from other species were clustered on separate branches. Thus, it indicated that *STM* may have differentiation among different species (**Figure S4**). The phylogenetic analysis confirmed that *BnaA09.STM* and *BnaC09.STM* clustered with *BraA09.STM* and *BolC09.STM* and implied that these two *BnaSTM* homologs might have conserved yet redundant functions, originating from two diploid progenitors.

### 3.2 Expression analysis of the *BnaSTM*

**Figure 1:**
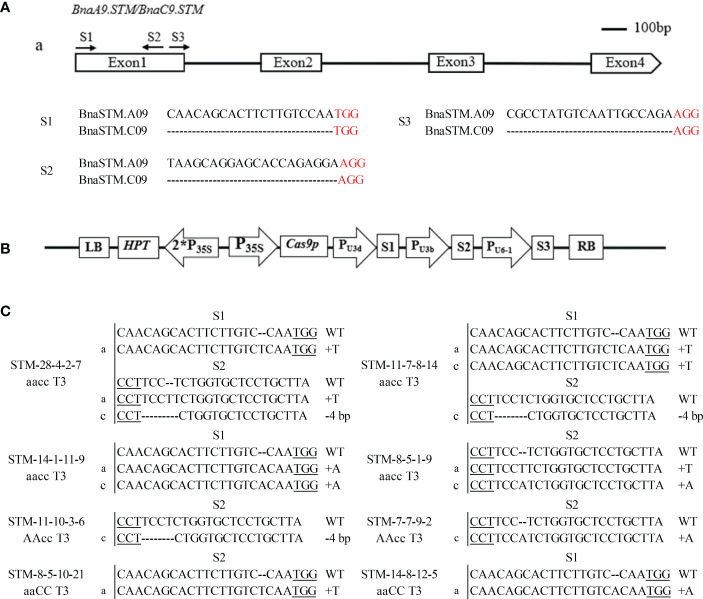
Expression pattern of *BnaSTM* in rapeseed. **(A)** qRT-PCR for *BnaSTM* transcripts in different developmental tissues and stages of J9707. The expression was compared to that of the control *BnACT7* gene. SAM, shoot apical meristem. **(B)** Fragments Per Kilobase of exon model per Million mapped fragments (FPKM) values for *BnaSTM* in different developmental tissues of ZS11. The data are presented as means ± SE (n ≥ 3); *LSD* was used to compare the expression of two copies of *BnaSTM* (**P > 0.01).

To understand the expression pattern of *BnaSTM*, different tissues, including roots, stems, leaves, flowers, SAM, buds, and 7 days after pollination (contain embryos at the globular stage) seeds, were collected from J9707 for qRT-PCR analysis. It showed that the *BnaSTM* was highly expressed in SAM and stem (**Figure 1A**). Although the expression levels of *BnaA09.STM* and *BnaC09.STM* has the same trend in different tissues, the expression level of *BnaC09.STM* was significantly higher than *BnaA09.STM*.

Using the data of a recent public RNA-seq data (http://yanglab.hzau.edu.cn/BnTIR) in ZS11 (a reference genome of rapeseed), expression profile of *BnaSTM* in ZS11 is showed in **Figure 1B**. Among different tissues, *BnaA09.STM* and *BnaC09.STM* has the highest expression levels in stem and bud. Moreover, the expression level of *BnaC09.STM* was significantly higher than that of *BnaA09.STM*, which was consistent with the differential expression of the two homologous copies of *BnaSTM* in J9707.

### 3.3 Creation of CRISPR/Cas9-targeted mutations in *BnaSTM*

**Figure 2:**
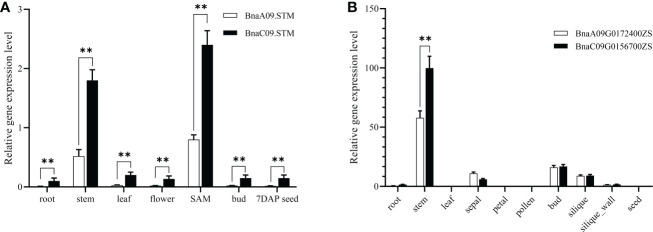
CRISPR/Cas9-induced mutants of *BnaSTM* in *B. napus*. **(A)** The *BnaSTM* gene model includes four exons (box) separated by three introns (represented by the solid line). The vertical line in the gene model indicates the target site, and the arrow indicates the sgRNA direction. The target sequences are shown with the protospacer adjacent motif (PAM) underlined. **(B)** The CRISPR/Cas9 construct comprised of a hygromycin resistance cassette (consisting of the hygromycin phosphotransferase coding sequence driven by the cauliflower mosaic virus 35S promoter); a Cas9 expression cassette (comprising the sequence encoding Cas9 driven by P35S); three sgRNAs (S1-S3) driven by the U3d, U3b and U6-1 promoter from Arabidopsis. **(C)** Sequences at the sgRNA target sites of *BnaSTM* homozygous mutants in the T_3_ generation. The PAM is underlined, and nucleotide indels are marked in red, with details labeled at right, ‘a’ and ‘c’ represent the mutated alleles of the target gene on *BnaA09.STM* and *BnaC09.STM*, respectively. ‘aaCC’, ‘AAcc’ and ‘aacc’ represent homozygous mutations of the target gene in *BnaA09.STM*, *BnaC09.STM* and both copies, respectively.

To generate the Cas9-induced knockout mutations in both copies of *BnaSTM*, three sgRNAs, viz. sgRNA1 (S1), sgRNA2 (S2), and sgRNA3 (S3), were designed using the CRISPR-P program (Lei et al., 2014). All of these sgRNAs were designed to target the first exon of *BnaSTM* and ensure the success rate of gene disruptions altering the reading frame. The sgRNAs precisely matched all the *BnaSTM* copies (**Figure 2A**). Based on the CRISPR/Cas9 multiple genome editing vectors as previously described by Yang et al. (2018), CRISPR/Cas9 constructs containing these three sgRNAs were generated, in which Cas9 was driven by the 35S promoter (Yang et al., 2018) (**Figure 2B**).

The resulting CRISPR/Cas9 construct was transformed into J9707 strain using *Agrobacterium*-mediated transformation. The targeted mutations in all T_0_-positive transgenic plants were identified using Sanger DNA sequencing of PCR products. Five lines with different targeted mutation types were identified in T_0_-positive transgenic plants (**Table 1**).

**Table 1:** Genotypic analysis of *BnaSTM* mutants and their transmission from T_0_ to T_1_, T_2_, and T_3_ generations.

Plant ID	Generation	Genotype at targets of BnSTM.A09			Genotype at targets of BnSTM.C09			Fused cotyled on petiole (Y/N)
		S1	S2	S3	S1	S2	S3	
STM-8	T0	Hetero (+1 bp)	Homo (+1 bp)	WT	Hetero (+1 bp)	Hetero (+1 bp)	WT	N
STM-8–5	T1	Hetero (+1 bp)	Homo (+1 bp)	WT	Hetero (+1 bp)	Hetero (+1 bp)	WT	N
STM-8–5–1	T2	WT	Homo (+1 bp)	WT	WT	Homo (+1 bp)	WT	Y
STM-8–5–1–9	T3	WT	Homo (+1 bp)	WT	WT	Homo (+1 bp)	WT	Y
STM-8–5–10	T2	Hetero (+1 bp)	Homo (+1 bp)	WT	Hetero (+1 bp)	WT	WT	N
STM-8–5–10–21	T3	WT	Homo (+1 bp)	WT	WT	WT	WT	N
STM-8–5–10–6	T3	WT	Homo (+1 bp)	WT	Hetero (+1 bp)	WT	WT	N
STM-28	T0	Hetero (+1 bp)	biallelic (+1 bp, –2 bp)	WT	Hetero (+1 bp)	Hetero (-4 bp)	WT	N
STM-28–4	T1	Hetero (+1 bp)	biallelic (+1 bp, –2 bp)	WT	WT	Hetero (-4 bp)	WT	N
STM-28–4–2	T2	Homo (+1 bp)	Homo (+1 bp)	WT	WT	Homo (-4 bp)	WT	Y
STM-28–4–2–7	T3	Homo (+1 bp)	Homo (+1 bp)	WT	WT	Homo (-4 bp)	WT	Y
STM-11	T0	Hetero (+1 bp)	Hetero (+1 bp)	WT	Hetero (+1 bp)	Hetero (-4 bp)	WT	N
STM-11–7	T1	Hetero (+1 bp)	Hetero (+1 bp)	WT	Hetero (+1 bp)	Homo (-4 bp)	WT	N
STM-11–7–8	T2	Homo (+1 bp)	WT	WT	Homo (+1 bp)	Homo (-4 bp)	WT	Y
STM-11–7–8–14	T3	Homo (+1 bp)	WT	WT	Homo (+1 bp)	Homo (-4 bp)	WT	Y
STM-11–10	T1	Hetero (+1 bp)	WT	WT	WT	Homo (-4 bp)	WT	N
STM-11–10–3	T2	WT	WT	WT	WT	Homo (-4 bp)	WT	N
STM-11–10–3–6	T3	WT	WT	WT	WT	Homo (-4 bp)	WT	N
STM-14	T0	Hetero (+1 bp)	WT	WT	Hetero (+1 bp)	WT	WT	N
STM-14–1	T1	Hetero (+1 bp)	WT	WT	Homo (+1 bp)	WT	WT	N
STM-14–1–11	T2	Homo (+1 bp)	WT	WT	Homo (+1 bp)	WT	WT	Y
STM-14–1–11–9	T3	Homo (+1 bp)	WT	WT	Homo (+1 bp)	WT	WT	Y
STM-14–8	T1	Homo (+1 bp)	WT	WT	Hetero (+1 bp)	WT	WT	N
STM-14–8–12	T2	Homo (+1 bp)	WT	WT	Hetero (+1 bp)	WT	WT	N
STM-14–8–12–5	T3	Homo (+1 bp)	WT	WT	WT	WT	WT	N
STM-7	T0	WT	Hetero (+1 bp)	WT	WT	Homo (+1 bp)	WT	N
STM-7–7	T1	WT	Hetero (+1 bp)	WT	WT	Homo (+1 bp)	WT	N
STM-7–7–9	T2	WT	Hetero (+1 bp)	WT	WT	Homo (+1 bp)	WT	N
STM-7–7–9–2	T3	WT	WT	WT	WT	Homo (+1 bp)	WT	N

Hetero, heterozygous; Homo, homozygous; WT, wild type. “−” and “+” indicate the deletion and insertion of the indicated number of nucleotides, respectively. All other targets are WT except the indicated target.

To obtain stable lines with targeted mutations, these T_0_ editing lines of *BnaSTM* were self-pollinated, producing T_1_, T_2_ and T_3_ progeny. The targeted mutations of progeny from T_0_ lines were verified by Hi-TOM sequencing analysis of the target sites. The results showed that allelic mutations in the T_0_ editing lines were transmitted to the following generations, indicating the stable germ-line transmission of Cas9-induced mutations in rapeseed (**Table 1**). A variable number and type of homozygous mutants with different allelic combinations of these *BnaSTM* copies were detected, including 7 *BnaA09.STM* single mutants, 8 *BnaC09.STM* single mutants and 8 *Bnastm* double mutants (**Figure 2C**; **Table 1**). All of the detected homozygous mutations at the target sites within *BnaSTM* were predicted, causing frameshifts and resulting in nonfunctional proteins (**Figure S5**).

### 3.4 Targeted mutations in *BnSTM* lead to fusion of the cotyledon petiole in seedling

**Figure 3:**
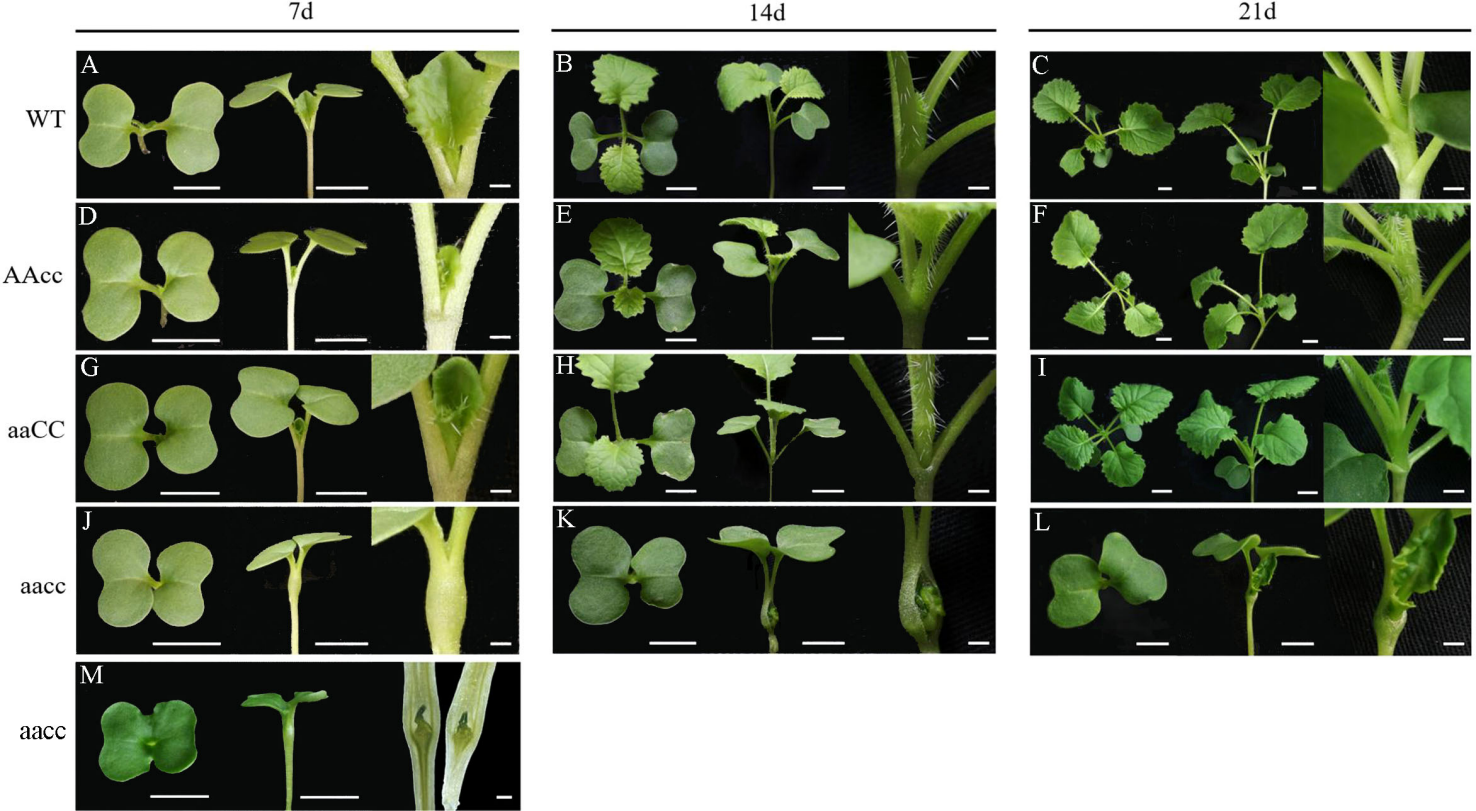
Phenotype of BnaSTM mutant in the T2 generation. **(A–C)** Top view and side view of WT seedlings at 7, 14, and 21 DAG, respectively; **(D–F)** Top view and side view of AAcc seedlings at 7, 14, and 21 DAG, respectively; **(G–I)** Top view and side view of aaCC seedlings at 7, 14, and 21 DAG, respectively; **(J–L)** Top view and side view of aacc seedlings at 7, 14, and 21 DAG, **(M)** Part of the aacc material has an extreme cup-shaped cotyledon phenotype. ‘aaCC’, ‘AAcc’ and ‘aacc’ represent homozygous mutations of the target gene in BnaA09.STM, BnaC09.STM and both copies, respectively. Top view and side view scale bars = 1 cm, the Partial view scale bars = 0.01 cm. Section scale bars = 0.5 mm.

To dissect the functions of the *BnaSTM*, some homozygous mutant T_2_ and T_3_ lines with different frameshift mutations (**Figure S5**; **Table 1**) were chosen for subsequent phenotypic characterization. As expected, all the double mutants could produce a visible knockout phenotype (**Figure 3**), i.e., a fused cotyledon petiole (the base of the two petioles fused; **Figure 3J**) or cup-shaped cotyledon (two petioles were fully fused, and the two cotyledons were also fused together; **Figure 3M**) at 7 days after germination (DAG), while the single mutants showed a comparable phenotype to that of the WT (**Figures 3D, G**). The longitudinal section of the cotyledon fusion site clearly showed that true leaves were wrapped inside of the *Bnastm* double mutants (**Figure 3M**). Eventually, the true leaves of *Bnastm* double mutants broke out from the cotyledon fusion site about 14 DAG (**Figure 3K**).

**Figure 4:**
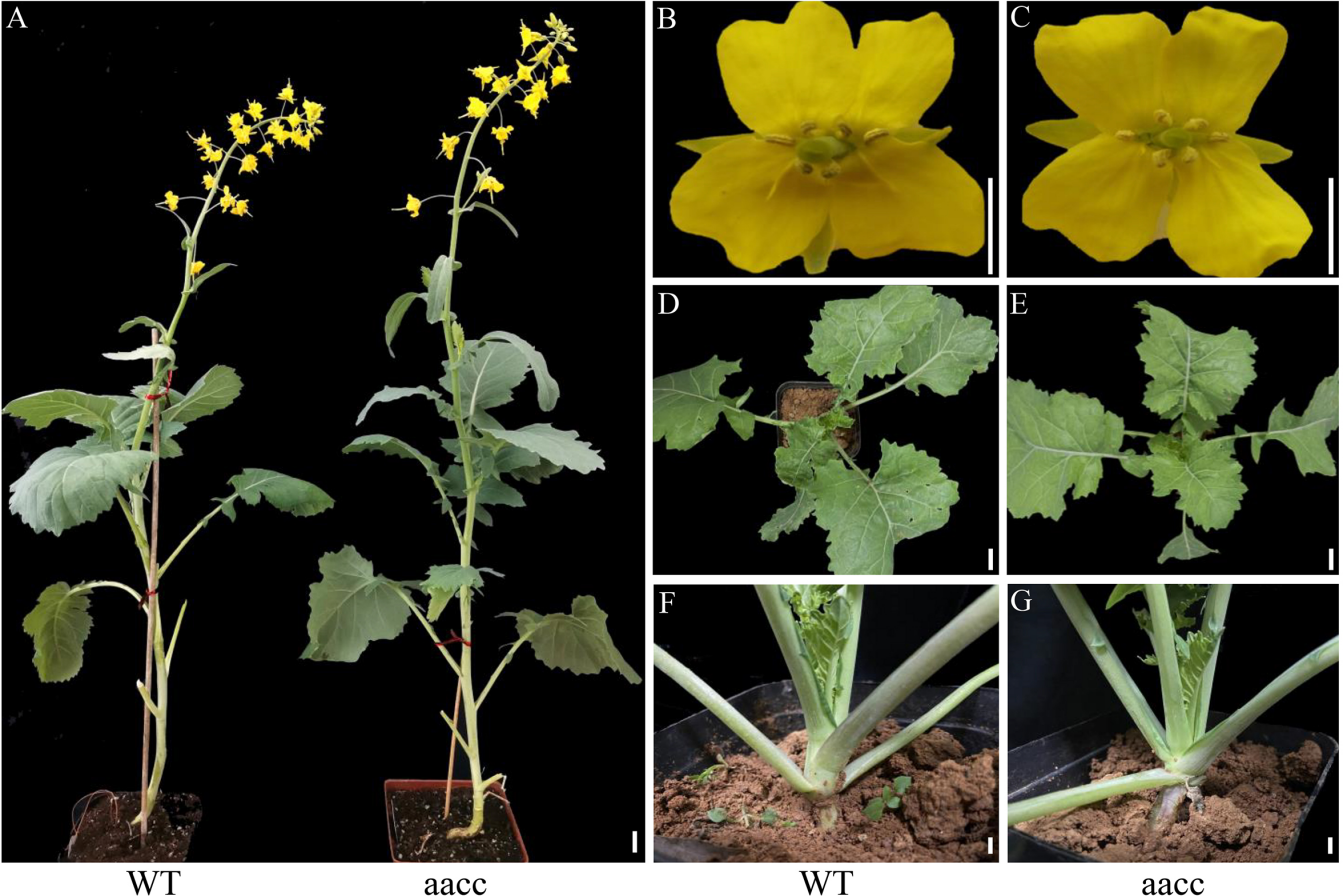
Phenotype of BnaSTM mutant in the T_3_ generation. **(A)** Representative plants at the bolting stage of WT and aacc. ‘aacc’ represent homozygous mutations of the target gene in *BnaSTM* both copies. **(B, C)** flowers of WT and aacc. **(D, E)** 40-day old plants of WT and aacc. **(F, G)** 40-day old plants of WT and aacc. The view Scale bars = 1 cm.

From 7 to 21 DAG, the development of mutant seedlings was significantly delayed than that of WT, which may ascribe the fusion of cotyledon petioles physically prevented the outgrowth of the new leaf. However, there was no significant difference between the mutants and WT in the late vegetative and reproductive growth period (**Figure 4**). Based on these results, we propose that *BnaSTM* function mainly in early seedling development and *BnaA09.STM* and *BnaC09.STM* has a redundant function, and the normal expression of *BnaSTM* in *B. napus* is of great significance for the normal separation of cotyledons.

### 3.5 Cytological observation of SAM in early stage

**Figure 5:**
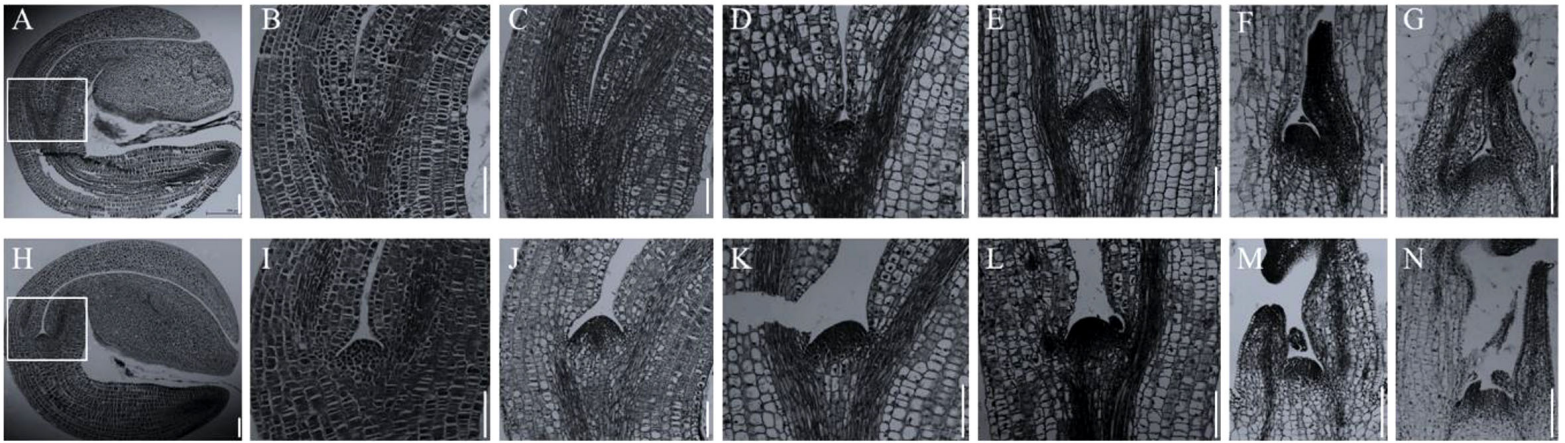
Cytological observation of early stem apical development. **(A–G)** SAM development of *Bnastm* double mutant seeds on days 1, 2, 3, 4, 6, and 8 after germination; **(H–N)** WT observation on SAM development of mutant seeds on days 1, 2, 3, 4, 6, and 8 after germination; **(B, I)** In **(A, H)**, respectively, the SAM is magnified by 2.5 times; **(C, J)** Second day after germination; **(D, K)** Third day after germination; **(E, L)** Fourth day after germination; **(F, M)** Sixth day after germination; **(G, N)** Eighth day after germination. Bar = 164 µm.

In Arabidopsis, the *stm* mutants have abnormal SAM throughout the growth period (Scofield and Murray, 2006). However, in allotetraploid *B. napus*, the abnormality of SAM in *Bnastm* double mutant only occurred during early germination process (**Figure 5**). The *Bnastm* mutant had no SAM in mature embryo in comparison with WT (**Figures 5A, B, H, I**). On the third day of germination, a small number of cells appeared at the top of the stem to form a small SAM (**Figure 5D**). On the fourth day of germination, the development of SAM in the *Bnastm* double mutant was almost normal, whereas its size was slightly smaller than that of WT (**Figure 5E**).

Additionally, in comparison with WT, the true leaves formation in *Bnastm* double mutant were delayed. On the sixth day of germination, the *Bnastm* double mutant had only one true leaf, while the WT had two visible true leaves (**Figures 5F, M**). On the eighth day of germination, the true leaves of the *Bnastm* double mutant were tightly packed inside (**Figure 5G**). However, the true leaves in WT were not packed together, and there was a large gap between two separated cotyledon petioles (**Figure 5N**). These results were differed from the phenotype of *stm* mutant in *A. thaliana*, indicating that there were more complex mechanisms in allotetraploid *B. napus*, for the regulation of SAM.

### 3.6 Off-target activity of CRISPR/Cas9 in T_0_ and T_1_ transgenic *B. napus* plants

To determine whether the fusion of the cotyledon petiole phenotype was resulted from mutations of off-target sites in the edited lines, off-target effects were explored in the edited plants by the CRISPR-P program (Lei et al., 2014). These potential off-target sites are listed in **Table S2**. S1 and S2 have 7 and 10 putative off-target sites (**Table S2**), respectively, and all predicted off-target genes and target sites had four base differences, making it difficult to edit effectively, and all predicted off-target genes do not belong to the *KNOX* gene family. These findings demonstrated that the sgRNAs have high specificity for targeting the *BnaSTM* in *B. napus*.

### 3.7 *BnaSTM* regulates the expression of genes involved in SAM boundary formation and organogenesis

Given the central importance of *STM* in SAM and cotyledon petiole development, we performed RNA sequencing on *Bnastm* double mutants (aacc) and the corresponding WT using fused cotyledon petiole containing the SAM of the 7-days-old-seedlings. A total of 69,134 genes were expressed in seedlings during the same period and were included in the subsequent analysis. Pearson’s correlation coefficient between any two of the three biological replicates was very high (R = 0.93–0.98) in both mutant and WT, which indicated that the transcriptome sequencing data used in this study were highly reliable (**Figure S6**; **Table S3, S4**).

Because the expression of many genes in SAM is not high and the expression change is not obvious, so | log_2_FC | > 1.5 is selected as the threshold of DEGs to expand the number of DEGs and find more genes that may be related to STM. Comparison of transcript abundances fused cotyledon petiole at 7 DAG uncovered 2,084 DEGs between each double mutant and its corresponding WT, which contained 1137 up-regulated genes as well as 947 down-regulated genes. The GO enrichment analysis showed that the two class of the positive regulation of organ growth and the polar auxin transport, which are important for meristem development, were significantly enriched in the up-regulated DEGs. The stem cell fate determination and the commitment were enriched in down-regulated DEGs (**Figure S7**, **S8**). And KEGG enrichment analysis of these identified DEGs showed that the down-regulated DEGs in the double mutants were significantly enriched in plant signal transduction pathway (**Figure S9**).

**Figure 6:**
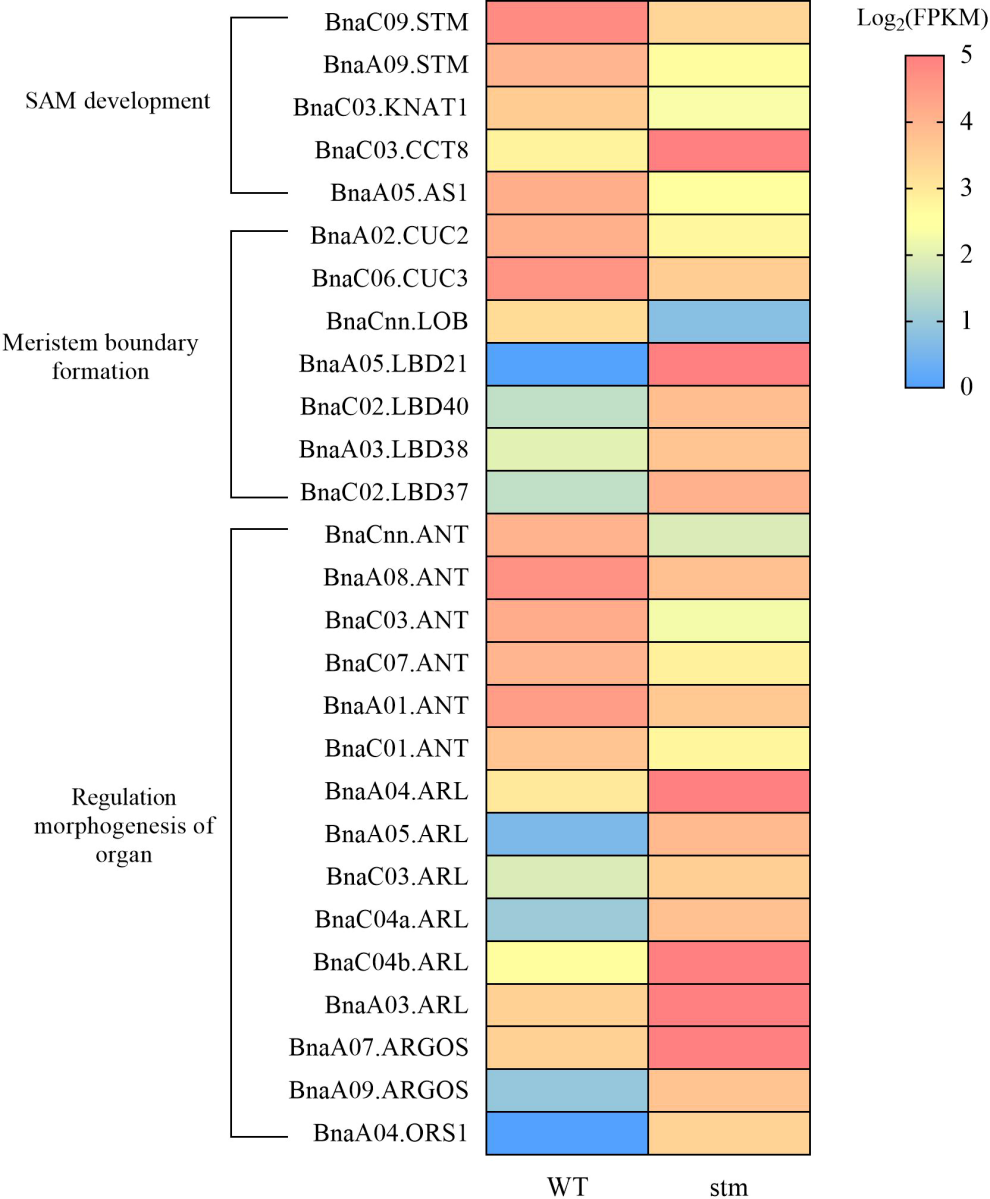
Heatmap showing the expression (FPKM) of important genes related to the development of shoot apical meristem (SAM) in double mutant and WT.

Among the DEGs, in the case of *KNOX* genes, except for *STM*, only the expression levels of one copy of *BnaKNAT1* were significantly down-regulated (**Figure 6**). In addition, the expression level of *BnaCCT8*, which controls the transport of STM between cells in the meristem, was down-regulated (**Figure 6**). For the genes related to lateral organ activation, such as *BnaYAB3*, *BnaAS1*, *BnaAS2* and *BnaJLO*, only the expression level of *BnaAS1* was down-regulated. Previous studies showed that *CLV3/WUS* maintains stem cell stability through feedback regulation in SAM (Schoof et al., 2000), however, the expression of *BnaCLV3* and *BnaWUS* as key genes in the pathway were not detected in WT and double mutant. Moreover, the *BnaCLV1*, *BnaCLV2* and *BnaCRN* were the receptor gene of *BnaCLV3* signal peptide, and their expression remained unchanged in the double mutant.

Notably, *BnaCUC2* and *BnaCUC3* were down-regulated to varying degrees (**Figure 6**). As we know that *CUCs* were involved in the formation of the SAM boundary (Kwon et al., 2006), The down-regulation of *CUCs* expression may lead to cotyledon petiole fusion. In addition, *LOB DOMAIN-CONTAINING (LBD)* gene family contain *LATERAL ORGAN BOUNDARIES (LOB)* domain, so *LBDs* can also regulate the formation of plant lateral organ boundary in plants (Xu et al., 2016). In our transcriptome data, the expression of *LBD* family genes showed irregular rise and fall, such as *BnaLBD40*, *BnaLBD21*, *BnaLBD38*, and other genes increased significantly, whereas, *BnaLOB* gene expression decreased significantly.

*AP2-LIKE ETHYLENE-RESPONSIVE TRANSCRIPTION FACTOR* (*ANT*) can maintain the meristematic competence of cells and consequently sustains expression of cell cycle regulators during organogenesis, thus controlling the final size of each organ by controlling their cell number (Horstman et al., 2014). *AUXIN-REGULATED GENE INVOLVED IN ORGAN SIZE* (*ARGOS*)*, ARGOS-LIKE PROTEIN* (*ARL*) *and ORGAN SIZE RELATED 1* (*ORS1*) regulate the formation of organs by regulating the expression of *ANT* (Hu et al., 2003; Hu et al., 2006; Feng et al., 2011). RNA-seq data shows that in the double mutant, the expression of six *BnaANT* copies is significantly down-regulated, and some copies of *BnaARGOS*, *BnaARL* and *BnaORS1* are also differentially expressed (**Figure 6**). Thus, decrease of the genes in meristem boundary formation might be responsible for the fused cotyledon petiole (**Figure 3J**) and cup-cotyledon phenotype (**Figure 3M**).

As *AtSTM* is engaged in CK and GA metabolism (Jasinski et al., 2005; Yanai et al., 2005), we also dive into our transcriptome data for CK and GA pathways, however, to our surprise, although plant hormone signal transduction pathway was significantly enriched in down-regulated gene sets (**Figure S9**), the expression of related genes which were previously shown to be involved in SAM regulation (Jasinski et al., 2005), such as *GA20ox1*, did not change. Only *BnaARF5* were significantly down-regulated. This is probably the reason why *Bnastm* double mutant plants could develop to normal plants while in Arabidopsis *stm* mutant plants had severer defects in plant development (Jasinski et al., 2005).

To verify the reliability of the RNA-seq data, 21 genes in the seedling were selected for qRT-PCR verification analysis. These genes included 12 genes related to SAM development, and 9 were randomly selected DEGs (**Table S5**). The linear regression analysis showed that the measured correlation coefficient between the two sets of gene transcription levels was very high (R = 0.884; **Figure S10**), further confirming the reliability of the RNA-seq data.

The results showed that *BnaSTM* plays an important role in the development of SAM. Its expression can cause changes in the formation of SAM boundaries as well as early organogenesis. These results indicated that *BnaSTM* has more complex regulatory networks and mechanisms in *B. napus*.

### 3.8 *Bnastm* altered expression of genes in FA and glucosinolate biosynthesis pathways in the seedling but not the of FA and glucosinolate content in the seeds

Interestingly, the expression levels of genes related to fatty acid synthesis and oleic acid synthesis were significantly up-regulated. For instance, one of the three copies of *BnaFAD2* and one copies of *BnaAAC1* was up-regulated, which are the key genes for fatty acid synthesis (**Table S6**). In addition, based on the KEGG enrichment analysis, glucosinolate biosynthesis pathway was significantly enriched in both up- and down-regulated gene sets (**Figure S9**; **Table S7**). The expression level of genes *BnaBCAT4*, *BnaMAM1*, *BnaIPMDH1* and *BnaIIL1* regulating the side-chain elongation of glucosinolate was significantly down-regulated, and the expression level of *BnaSOT18* gene regulating the synthesis of aliphatic and indole glucosinolate core structure was significantly up-regulated.

**Figure 7:**
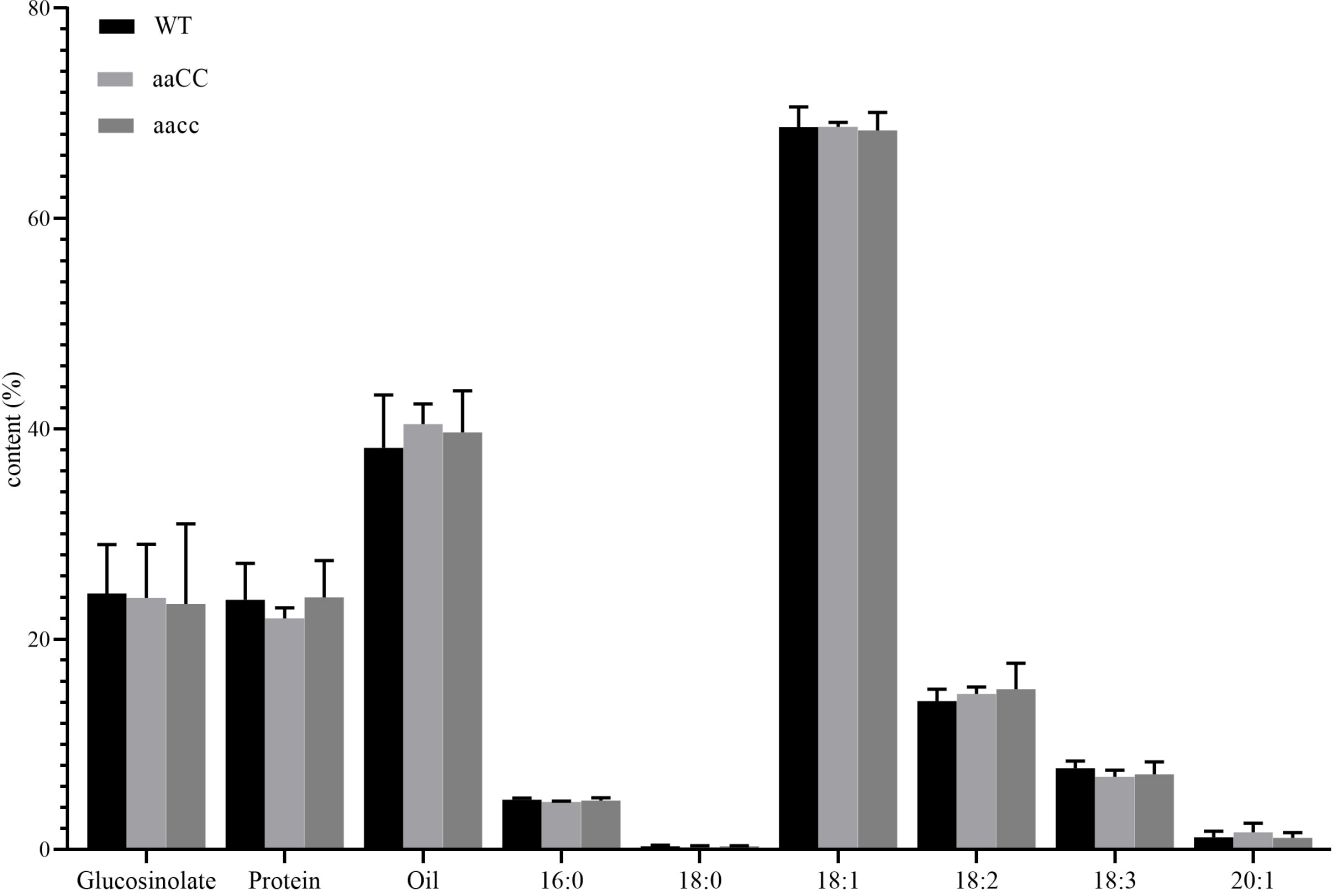
Assay of glucosinolate content, protein content and FA composition in seeds of BnaSTM single (aaCC) and double (aacc) mutants and WT. Three biological replicates were performed, and means ± SE are shown (Student’s t-test).

Fatty acid composition and content, protein content and glucosinolate content are important traits in rapeseed. The composition and content of fatty acids determine the yield and quality of oil, while the content of protein determines the quality of rapeseed meal. Glucosinolates are anti-cancer and antioxidant biochemical compounds that can protect plants from insects and microorganisms (Halkier and Gershenzon, 2006). In *B. napus*, reducing its content in seeds and increasing its content in leaves and other tissues is also one of the goals of rapeseed breeding. Through phenotypic and genotypic analysis, it was found that aaCC and aaCC mutants had almost no difference from WT at early seedling stage (**Figure 3**; **Table 1**), and only the double mutants had fused cotyledons. Therefore, we have reason to believe that both copies of *STM* in *B. napus* have redundancy function. Therefore, we only selected mature seeds of aaCC and aacc for the analysis of the fatty acid and glucosinolate contents. To our surprise, neither fatty acid nor glucosinolate contents were changed (**Figure 7**). The total oil content and the three major unsaturated fatty acids: oleic acid (C18:1), linoleic acid (C18:2) and linolenic acid (C18:3) were not significantly changed compared with WT.

## 4 Discussion

The *KNOX* transcription factor *STM* is essential during shoot apical meristem development. In *Arabidopsis thaliana*, *STM* regulates hormone synthesis and response. It also coordinates the organ formation and differentiation, leaf type, meristem, and the establishment of organ boundary region (Spinelli et al., 2011; Kamiuchi et al., 2014; Balkunde et al., 2017). Therefore, it is also crucial to explore the mechanism of this gene in the important oil crop *B. napus*.

### 4.1 Application of CRISPR/Cas9 system in this experiment

Plant genome editing technology depends on plant genetic transformation to a large extent. Compared with other crops, the transformation efficiency of most *B. napus* is relatively low. For example, Braatz et al. used the Cas9/sgRNA system to conduct gene editing with the spring cultivar Haydn as the transformation recipient, and its first-generation transformation rate was only 0.9% (Braatz et al., 2017). Therefore, this experiment selected a pure *B. napus* line J9707, which is a good transformation receptor, and the transformation rate is between 66.7% and 92.5% (Yang et al., 2018). At the same time, Hi-tom, a high-throughput sequencing method, is also used to quickly identify genotypes (Yang et al., 2017).

For using CRISPR/Cas9 system, off-target locus check is an important step to perform. According to the results of the editing and off-target identification of other genes in *B. napus* by the research teams through CRISPR/Cas9 system (Yang et al., 2017; Yang et al., 2018), when the predicted number of base differences between the target locus and the off-target locus is greater than or equal to four, there is no off-target. Moreover, the predicted genes at the off-target locus in this experiment are not related to the development of SAM. Therefore, in the present study, we did not verify the off-target locus by PCR.

What’s more, as an allotetraploid, many genes in *B. napus* have copy redundancy. The *BnaSTM* in the present study only has two copies, we used a CRISPR/Cas9 system that can mutate multiple target locus at the same time in *B. napus* (Yang et al., 2017), which showed good editing efficiency for both copies, and this system was successfully applied to *BnaCLV3*, which also contained two copies (Yang et al., 2018). However, as for the genes with greater number of copies, it is very difficult to edit all copies at the same time.

### 4.2 The phenotypes of *Bnastm* mutant were different from *Atstm*

In the present study, CRISPR/Cas9 technology was successfully employed to target mutations in *BnaSTM*. The induced mutations can be stably inherited to progeny. The *Bnastm* double mutant showed a fused cotyledon petiole phenotype at the seedling stage (**Figure 3J**), which was similar to the *stm-1* (strong mutant allele) in the model plant Arabidopsis (Nidhi et al., 2021). In Arabidopsis, the fusion was restricted to the region from the base to the middle part of the petioles and did not extend to the cotyledon ([]Clark, 1996; Long et al., 1996; Aida et al., 1999; Belles-Boix et al., 2006; Nidhi et al., 2021). However, one of the *Bnastm* double mutant seedling had a cup-shaped cotyledon phenotype (**Figure 3M**), which was not observed in *stm* mutants in Arabidopsis, but in double mutant with *KNAT6* and *CUC2* (Belles-Boix et al., 2006). This indicates that early boundary regulation of *STM* in *B. napus* is more complex.

In addition to the seedling phenotype, *Bnastm* also had great differences in the SAM, leaves and inflorescence development compared with *Atstm*. The cytological observation revealed that SAM was not observed in the mature embryo of double mutant (**Figure 5A**) which was the same as in Arabidopsis (Clark, 1996). During seed germination process, *BnaSTM* gradually restored SAM after 3 days of germination with no significant difference from WT (**Figure 5D**). The true leaves which developed later gradually broke wrapped state and resumed normal growth in the late vegetative growth period (**Figure 3L**). The growth and development of the double mutant in the seedling stage were slightly slower than WT (**Figure 3K**). In addition to physical oppression, it may also be that SAM formation in *Bnastm* double mutants delayed compared to the WT one, resulting in delayed expression of genes that regulate stem cell differentiation. The results showed that SAM could develop normally in WT, and that the genes regulating stem cell differentiation could be expressed normally without *STM* mutation. Surprisingly, in the older stage of growth and development, there was no significant difference from the WT (**Figure 4**). However, in Arabidopsis. the severe mutant *stm-1* in Arabidopsis lack an embryonic shoot meristem and could not produce normal leaves and inflorescence (Clark, 1996; Nidhi et al., 2021), while a weak allele *stm-2* could produce leaves and inflorescences that were not indeterminate structures as in WT plants (Clark, 1996). This implies that there may also be functionally redundant genes with *BnaSTM* in *B. napus* for functional compensation or *BnaSTM* has more complex regulatory networks and mechanisms in *B. napus* compared with *A. thaliana*.

### 4.3 *BnaSTM* is mainly involved in the shoot meristem boundary formation and organogenesis

According to transcriptome results, in the *KNOX I* gene family, except *BnaSTM*, only the expression level of *BnaKNAT1* was significantly reduced in the double mutant. Research showed that *AS1* and *AS2* inhibit the expression of *KNOX I* genes including *KNAT1*, *KNAT2*, *KNAT6* and *STM* (Semiarti et al., 2001). The down-regulation of *BnaAS1* expression in mutants may promote the up regulation of *BnaKNAT1*, *BnaKNAT2* and *BnaKNAT6* expression to compensate for the effect of *Bnastm* mutation. As the *BnaCCT8* functions in transporting the STM protein to maintain the homeostasis of stem cell (Xu et al., 2011), the down-regulating of the transporting of *STM* by *BnaCCT8* is probably the result of the down-regulation of *BnaSTM*, so that the homeostasis of stem cell was defected.

As we observed the fused cotyledon petiole in the seedling of *Bnastm* mutants, it was not surprising that genes involved in meristem boundary formation had significant changes in the transcriptome data compared with WT. *Bnastm* mutations significantly reduced the expression of *BnaCUC2* and *BnaCUC3*, which were known to be involved in the establishment of boundaries (Vroemen et al., 2003). However, the expression of other regulatory boundary genes was significantly increased in mutants, such as *BnaLBD* family genes. *BnaLBD* family genes were key regulators of lateral organ boundary set up and played essential roles in integrating developmental changes in response to phytohormone signaling or environmental cues (Xu et al., 2016). Although the specific functions of each gene in the *BnaLBD* gene family are still unclear, the expression patterns and functional domains of these genes suggest that up-regulation of these genes may compensate for the effects of *Bnastm* mutation (Clark et al., 1997) (**Figure 6**).

In addition to boundary formation genes, a sets of organogenesis related genes were significantly changed in *Bnastm* (**Figure 6**). *ANT* regulates cell proliferation and organ growth by maintaining the meristematic competence of cells during organogenesis (Mizukami and Fischer, 2000). *ANT* affects cell proliferation by regulating the expression of *Cyclin D3;1* (*CYCD3;1*) during organ growth (Hu et al., 2003). The expression of *BnaANT* decreased significantly (**Figure 6**), it can be speculated that there may be a certain regulatory mechanism between *BnaSTM* and *BnaANT*. When *BnaSTM* was knocked out, the expression of *BnaANT* gene was decreased, which together led to the smaller SAM (**Figure 5**) and true leaf size (**Figure 3**) compared with WT. Besides, increased expression of *BnAGROS*, *BnARL* and *BnORS1* was observed. *AGROS*, *ARL* and *ORS1* contain a conserved OSR motif which regulates organ growth and final organ size by affecting both cell proliferation and expansion, and *OSR1* regulates organ growth redundantly with *ARGOS* and *ARL* (Feng et al., 2011). In Arabidopsis, overexpression of *OSR1* prolonged the expression of *ANT* but not increase the expression level. Therefore, we hypothesized that in *B. napus*, the increase of the expression level of *BnaARGOS*, *BnaARL* and *BnaORS1* was the feedback mechanism of plant, which try to overcome the decrease of the expression of *ANT*.

Several studies have shown that *CLV/WUS* signaling pathway is an important pathway to maintain SAM homeostasis. According to previous studies, the inhibitory effect of *CLV* mutants on stem cell differentiation was weakened, leading to abnormal meristem development in plants and the formation of a multilocular angular phenotype (Clark et al., 1997; Song et al., 2021). It has been reported that *CLV3* and *WUS* are mainly expressed in the inflorescence meristem after bolting in Arabidopsis (Winter et al., 2007), and mainly expressed in flower buds and seeds in *Brassica* (Liu et al., 2021), and their expression levels are very low, which is consistent with the results in this study that *CLV3* and *WUS* are almost not expressed in WT and *stm* mutants.

According to reports in *Arabidopsis*, A ring of *AINTEGUMENTA* gene expression distinguishes the peripheral domain, from which the cotyledons arise, from the central domain, where the meristem will form. *CUCs*, *STM* and *KNAT6* are expressed in a stripe across the middle of the embryo between the cotyledon primordia (Barton, 2010). At present, there are few studies on *BnaSTM* in rapeseed and there is no report on how it is expressed in different regions in SAM, it may be possible to observe the specific expression region of *STM* in SAM of *B. napus* by constructing a fluorescent protein vector including *STM* and help us explain the specific mechanism of *STM* in *B. napus*.

### 4.4 Effect of *BnaSTM* on the biosynthesis of fatty acids and glucosinolate

Interestingly, the transcriptome data of 7-days-old seedling results showed that the expression of *BnaFAD2*, *BnaFAD3* and *BnaACC1* were up-regulated, which was related to fatty acid synthesis. According to a previous study, the *ACC1* participates in the cell proliferation and tissue mode in the development of SAM, which can act as a repressor of CK response (Baud et al., 2003). However, although the expression of *BnaFAD2* and *BnaFAD3* increased, the oil content and oil composition of mature seeds did not change significantly. Because *FAD2*, *FAD3* and other important genes regulating oil content are mainly expressed and play a role in silique and seed at the later stage of plant growth, these genes are differentially expressed in SAM in this experiment, and the expression period is far earlier than the period when seed start to form.

The same is true for genes related to glucosinolate biosynthesis (**Figure 7**; **Table S8**). The reason may be that the down-regulation of the expression of *BnaBCAT4*, as the starting gene regulating glucosinolate biosynthesis, leads to the down-regulation of the expression of genes in the MANs-IPMIs-IPMDHs cycle which regulating amino acid chain elongation of glucosinolate biosynthesis (Gao et al., 2014). *BnaSOT18*, as the key gene regulating the biosynthesis of aliphatic and indole glucosinolate, the up-regulation of its expression compensates for the effect of down-regulation of *BnaBCAT4* on glucosinolate biosynthesis.

Furthermore, our transcriptome data was acquired at the seedling stage, while the fatty acid content and the glucosinolate content were measured in the mature seeds. As in *B. napus*, we only observed the phenotype in seedling stage (**Figures 3**, **4**), but not in the adult plants (**Figure 5**), it could be speculated that *BnaSTM* is mainly function during the early organ formation but not in late plant development in *B. napus*.

In all, *STM* is an important gene regulating SAM in *B. napus*, have many unexplored functions. Further studies on identifying its function can help to understand the growth process of *B. napus* more clearly as well as a better understanding of other related genes.

## Data availability statement

The datasets presented in this study can be found in online repositories. The names of the repository/repositories and accession number(s) can be found in the article/**Supplementary Material**.

## Author contributions

YZ and CF conducted the study. YZ and CF designed the experiments. HL and XW performed the experiments. KY performed the bioinformatic analysis. XW and HL assisted in the material sampling. KY wrote the manuscript. HH, HL, OA, and CF helped in the revision of the manuscript. CF supervised the study. All authors contributed to the article and approved the submitted version.
