# Supplementary material for: Targeted mutagenesis of BnaSTM leads to abnormal shoot apex development and cotyledon petiole fusion at the seedling stage in Brassica napus L
Source: Front Plant Sci. 2023 Feb 14;14:1042430. doi: 10.3389/fpls.2023.1042430 (PMC9971503; doi:10.3389/fpls.2023.1042430)
Supplement: Supplementary file 1 [file DataSheet_1.docx]

*AtSTM* CTCTCTTCTGCTGCTTCCCTCTCCCCCATAACTATCACTATTTAGAATTTTCAATGTGGA 60

*BnaA09.STM* ------------------------------------------------------------ 0

*BnaC09.STM* ------------------------------------------------------------ 0

*AtSTM* AAAGGAAGCTGATTGTTGAAGCATAAATCCCGGGAGACCACTTTTGCATTTTCAAATAAT 120

*BnaA09.STM* ------------------------------------------------------------ 0

*BnaC09.STM* ------------------------------------------------------------ 0

*AtSTM* TAAATTAAACCATAGATACACACACACAGTTACTTACTCTTTTAGGGTTTCCCAATAAAT 180

*BnaA09.STM* ------------------------------------------------------------ 0

*BnaC09.STM* ------------------------------------------------------------ 0

*AtSTM* TTATAGTACTTTAATGTGTTTCATGATATTGATGATAAATGCTAGCTGTATTTACAATGG 240

*BnaA09.STM* ------------------------------------------------------------ 0

*BnaC09.STM* ------------------------------------------------------------ 0

*AtSTM* GGGCTCCTCTTTTTTTTTCTTTATGTTATTTTCATTTTTTATTTCTCATAGCTATATAGC 300

*BnaA09.STM* ------------------------------------------------------------ 0

*BnaC09.STM* ------------------------------------------------------------ 0

*AtSTM* AAAGCCAAAGTGAATAATAATACTAGTGAGAGAAAGAGAAGATGGAGAGTGGTTCCAACA 360

*BnaA09.STM* ---------------------ACTAGTGAGAGAAAGTGAAGATGGAGAGTGGTTCCAACA 39

*BnaC09.STM* ---------------------ACTAGTGAGAGAAAGTGAAGATGGAGAGTGGTTCCAACA 39

*************** ***********************

S1

*AtSTM* GCACTTCTTGTCCAATGGCTTTTGCCGGGGATAATAGTGATGGTCCGATGTGTCCTATGA 420

*BnaA09.STM* GCACTTCTTGTCCAATGGCTTTTGCCGGGGATAATAGTGATGGTCCGATGTGTCCTATGA 99

*BnaC09.STM* GCACTTCTTGTCCAATGGCTTTTGCCGGGGATAATAGTGATGGTCCGATGTGTCCTATGA 99

************************************************************

*AtSTM* TGATGATGATGCCGCCCATCATGACATCACATCAACATCATGGTCATGATCATCA----- 475

*BnaA09.STM* TGATGATGATGATGCCCGTCATAACATCACATCAACAACATCATGGTCATGATCAACAAC 159

*BnaC09.STM* TGATGATGATGATGCCCGTCATAACATCACATCAACAACATCATGGTCATGATCAACAAC 159

*********** **** **** ************** *** * * ** ****

*AtSTM* -ACATCAACAACAAGAACATGATGGTTATGCATATCAGTCACACCACCAACAAAGTAGTT 534

*BnaA09.STM* ATCAACATCAACAACAACATGATGGTTATGCATATCAGTCACACCACCAACAAAGTAGCT 219

*BnaC09.STM* ATCAACATCAACAACAACATGATGGTTATGCATATCAGTCACACCACCAACATAGTAGCC 219

** ** ****** ************************************* *****

*AtSTM*  CCCTTTTTCTTCAATCACTAGCTCCTCCCC---AAGGAACTAAGAACAAAGTTGCTTCTT 591

*BnaA09.STM*  CCCTTTTTCTTCAATCACTAACTCCTCCGTCTCAAGAAGCGAAGAACAAAGTTACATCTT 279

*BnaC09.STM*  TCCTTTTTCTTCAATCACTAACTCCTCCGTCTCAAGAAGCGAAGAACAAAGTTAGATCTT 279

******************** ******* *** * * ************ ****

S2

*AtSTM*  CTTCTTCTCCTTCCTCTTGTGCTCCTGCCTATTCTCTAATGGAGATCCATCATAACGAAA 651

*BnaA09.STM*  CTTGTTCTCCTTCCTCTGGTGCTCCTGCTTATTCTTTCATGGAGATCAATCACCAAAACG 339

*BnaC09.STM*  CTTGTTCTCCTTCCTCTGGTGCTCCTGCTTATTCTTTCATGGAGATCAATCACCAAAACG 339

*** ************* ********** ****** * ********* **** * *

*AtSTM*  TCGTTGCAGGAGGAATCAACCCTTGCTCCTCTTCCTCTTCTTCAGCCTCTGTCAAGGCCA 711

*BnaA09.STM*  AACTCCTCGCA---------GGAGGACTCAATCCCTGTTCTTCAGCCTCTGTCAAGGCCA 390

*BnaC09.STM*  AACTCCTCGCA---------GGAGGACTCAATCCCTGTTCTTCAGCCTCTGTCAAGGCCA 390

* * * * * * *** ***********************

S3

*AtSTM*  AGATCATGGCTCATCCTCACTACCACCGCCTCTTGGCCGCTTATGTCAATTGTCAGAAGG 771

*BnaA09.STM*  AAATCATGGGTCATCCTCACTACCACCGCCTCTTACTCGCCTATGTCAATTGCCAGAAGG 450

*BnaC09.STM*  AAATCATGGGTCATCCTCACTACCACCGCCTCTTGCTCGCCTATGTCAATTGCCAGAAGG 450

* ******* ************************ *** *********** *******

*AtSTM*  TTGG-------------------------------------------------------- 775

*BnaA09.STM*  TGATGGTCAAAGAAAACCCTTTTTACAAGAATTATACTATGAATGATTTCTTAACTAATG 510

*BnaC09.STM*  TGATGGTCAAAGAAAACCCTTTTTACAAGAATTATACTATGAATGATTTCTTAACTAATG 510

*

*AtSTM*  ------------------------------------------------------------ 775

*BnaA09.STM*  ATCCTTGTTTAATTGTCTACACAAAGAAGCAGAAGATTCTAGAAACCCTAGTTACGAAAC 570

*BnaC09.STM*  ATCCTTGTTTAATTGTCTACACAAAGAAGCAGAAGATTCTAGAAACCCTAGTTACGAAAC 570

*AtSTM*  ------------------------------------------------------------ 775

*BnaA09.STM*  TTTACCCTTTTTTTTACTTTTTTTCATAACCCTTTCATTACTTTGATGCTTTAGTTTGAT 630

*BnaC09.STM*  TTTACCCTTTCTTTTACT-TTTTTCATAACCCTTTCATTACTTTGATGTTTTAGTTTGAT 629

*AtSTM*  ------------------------------------------------------------ 775

*BnaA09.STM*  TTTGATGTCTCCTTTTTGTTTATTTCTTACTTAATCCAACAATATACCTAAACATGAAAT 690

*BnaC09.STM*  TTTGATGTCCCCTTTTTGTTTATTTCTTTCTTAATCCAACAATATACCTAAACATCAAAA 689

*AtSTM*  ------------------------------------------------------------ 775

*BnaA09.STM*  GAAATAAGTTTTCCTTAGTTGAGAAAGTTTCAAGAAGCTTGGAACAAATAAAAAACAGTT 750

*BnaC09.STM*  GAAATAAGTTTTCCTTATTTGAGAAAGTTTCAAGAAGCTTGGAACAAATAAAAAACAGTT 749

*AtSTM*  ------------------------------------------------------------ 775

*BnaA09.STM*  AAATTCAAGAACCCTATTTTCAAGAAGTGATCCAAAATATTATTGCGGGCTTTAAGATTA 810

*BnaC09.STM*  AAATTCAAGAACCCTATTTTCAAGAAATGATCCAAAATATTATTGCGGGCTTTAAGATTA 809

*AtSTM*  ------------------------------------------------------------ 775

*BnaA09.STM*  TATCATATATTTTCTTTGCTTGATCTCTATCTATGCTCATATATGTCATACCCACAAGAA 870

*BnaC09.STM*  TAACATATATTTTCTTTGCTTGATCT----CTATGCTCATATTTGTCATACCCACAAGAA 865

*AtSTM*  ------------------------------------------------------------ 775

*BnaA09.STM*  ACTTGACGATCCTTGCTTTTACATTTTAACTAAAGCGCTTTATGTCTATTATTTAATTAC 930

*BnaC09.STM*  ACTTGACGATCCATGCTTTTACATTTTAACTAAAGCGTTTTATGTCTGTTATTTAATTAC 925

*AtSTM*  -----------------AGCACCACCGGAGGTTGTGGCGAGGCTAGAGGAGGCATGCTCG 818

*BnaA09.STM*  AAAATTATCCAGGTGGGAGCTCCACCGGAAGTGCAGGCGAGGCTGGAAGAAACATGCTCG 990

*BnaC09.STM*  AAAATTATCCAGGTGGGAGCTCCACCGGAAGTGCAGGCGAGGCTGGAAGAAACATGCTCG 985

*** ******** ** ********* ** ** ********

*AtSTM*  TCTGCCGCAGCCGCTGCCGCATCTATGGGACCAACAGGATGTCTAGGTGAAGATCCAGGG 878

*BnaA09.STM*  TCTGCGGCGGCAGCCGCAGCGTCGATGGGACCCACAGGTTCTTTAGGTGAAGATCCAGGG 1050

*BnaC09.STM*  TCTGCGGTTGCCGCCGCAGCGTCGATGGGACCCACAGGTTCTTTAGGTGAAGATCCAGGG 1045

***** * ** ** ** ** ** ******** ***** * * *****************

*AtSTM*  CTTGATCAATTCATGGAAGCTTACTGTGAAATGCTCGTTAAGTATGAGCAAGAGCTCTCC 938

*BnaA09.STM*  CTTGATCAGTTCATGGAAGCGTACTGTGAAATGCTCGTTAAGTACGAGCAAGAACTCTCT 1110

*BnaC09.STM*  CTTGATCAGTTCATGGAAGCGTACTGTGAAATGCTCGTTAAGTACGAGCAAGAACTCTCT 1105

******** *********** *********************** ******** *****

*AtSTM*  AAACCTTTCAAGGAAGCTATGGTCTTCCTTCAACGTGTCGAGTGTCAATTCAAATCCCTC 998

*BnaA09.STM*  AAACCTTTTAAAGAAGCTATGGTCTTCCTTCAACACGTCGAGTGTCAATTCAAATCCCTC 1170

*BnaC09.STM*  AAACCTTTTAAAGAAGCTATGGTCTTCCTTCAACACGTCGAGTGTCAATTCAAATCCCTC 1165

******** ** ********************** ************************

*AtSTM*  TCTCTATCCTCACCTTCCTCT--------------------------------------- 1019

*BnaA09.STM*  TCTCTCTCCTCGCCTTCCTCCTTCTCTGGTTACTCTCTCTCTCTCTAAATCTCTGTATTT 1230

*BnaC09.STM*  TCTCTCTCCTCGCCGTCCTCCTTCTCTGGTTACTCTC----TCTCTAAATCTCTGTATTT 1221

***** ***** ** *****

*AtSTM*  ------------------------------------------------------------ 1019

*BnaA09.STM*  TCTTTAAGATATTGATTTATTCTTATAATGGCGTCATATTAAACGTAAGATGAGAAAAGT 1290

*BnaC09.STM*  TCTTTAAGATATTGATTTATTCTTATAATGGCGTCATATTAAACGTAAGATAAGAAAAGT 1281

*AtSTM*  ------------------------------------------------------------ 1019

*BnaA09.STM*  GTTGCTTTTTTCCCTTTGTGGGCTTTAAGAAAGATTTTTCCTTTTTCTTGTGTTAGTGCC 1350

*BnaC09.STM*  GTTGCTTTTTTCCCTTTGTGGGCTTTAAGAAAGATTTTTCCTTTTTCTTGTGTTAGTGCC 1341

*AtSTM*  ------------------------------------------------------------ 1019

*BnaA09.STM*  CTTAATGCTTGTTGCTAGGGTTAGTGTCATTGTGTCAGATTGTAATATGTAGCTTCTAGG 1410

*BnaC09.STM*  CTTAATGCTTGTTGCTAGGGTTAGTGTCATTGTGTCAGATTGTAATATGTAGCTTCTAGG 1401

*AtSTM*  ------------------------------------------------------------ 1019

*BnaA09.STM*  TTTACGTGTACAATCGATCTATACATGTGTGTATGTCCATGTTTTACTGAAAGACAAGCT 1470

*BnaC09.STM*  TTTACGTGTACAATCGAT-TATACATATGTGTATGTCCATGTTTTACTGAAAGACAAGCT 1460

*AtSTM*  ------------------------------------------------------------ 1019

*BnaA09.STM*  TAAAAACAATATTTGAAGTTTCTTTTTCTGAAGTGAATTTATTTTGCTTGGTACAACAAC 1530

*BnaC09.STM*  TAAAAACAATATTTGAAGTTTCTTTTTCTGAAGTGAATTTATTTTGCTTGGTACAACAAC 1520

*AtSTM*  ------------------------------------------------------------ 1019

*BnaA09.STM*  ATTCACTGTTCTTGTCTCTTTCGTCGCCGTTAGATTTATTAAATTTATTGCGTCAAAAAG 1590

*BnaC09.STM*  ATTCACTGTTCTTGTCTCTTTCGTCGCCGTTAGATTTATTAAATTTATTGCGTCAAAAAG 1580

*AtSTM*  ------------------------------------------------------------ 1019

*BnaA09.STM*  AATATCTGGATCTAGAATTTGTTCATCCATAGATCTATTGCTCAATATAGACATACACAT 1650

*BnaC09.STM*  AATATCTGGATCTAGAATTTGTTCATCCATAGATCTATTGCTCAAAAAA----------- 1629

*AtSTM*  ------------------------------------------------------------ 1019

*BnaA09.STM*  GTAAAAAACGTATTATCTCATATACATATGCACTAGTGTCATATCACTAGTGTCTTGTCT 1710

*BnaC09.STM*  --------CGTATAATCTCATATACGTATGCACTAGTGTCATATCACTAGTGTCTTGTCT 1681

*AtSTM*  ------------------------------------------------------------ 1019

*BnaA09.STM*  TCACGAGTGTGGTTAAGCTTGTCTACACACACACCTACTTGCTC---------------- 1754

*BnaC09.STM*  TCACGAGTGTGGCTAAGCTTGTCTACACACACACCTACTTGCTCAACTTATTTCTTGATG 1741

*AtSTM*  ------------------------------------------------------------ 1019

*BnaA09.STM*  --------------------AACTTATTTCTTTGAATTGTTTGGATCACTAGCTCATTAT 1794

*BnaC09.STM*  ACAAAAATAAAAAGAAGCTCAACTTATTTCTTTGAATTGTTTGGATCACTAGCTCATTAT 1801

*AtSTM*  ------------------------------------------------------------ 1019

*BnaA09.STM*  ACTTGATTCCACTTAGCCGGTTTTGGTGATAACTTTGTCTTGCTTTCGTTTTGTTTTGTT 1854

*BnaC09.STM*  ACTTGATTCCACTTAGCCGGTTTTGGTGATAACTTTGTCTTGCTTTCGTT-----TTGTT 1856

*AtSTM*  ---------------TTCTCCGGTTATGGAGAGACAGCAATTGATAGGAACAATAATGGG 1064

*BnaA09.STM*  TTGGCATGATGTATCGAAGTAGGTTATGGAGAGGCAGCTATTGAGAGAAACAACAATGGG 1914

*BnaC09.STM*  TTGGCATGATGTATCGAAGTAGGTTATGGAGAGGCAGCTATTGAGAGAAACAACAATGGG 1916

************ **** ***** ** ***** ******

*AtSTM*  TCATCCGAGGAAGAAGTCGATATGAACAATGAATTTGTAGATCCACAAGCTGAGGATAGA 1124

*BnaA09.STM*  TCATCTGAGGAAGAAGTCGATATGAACAATGAATTTGTAGATCCGCAGGCAGAAGATAGG 1974

*BnaC09.STM*  TCATCTGAGGAAGAAGTCGATATGAACAATGAATTTGTAGATCCGCAGGCAGAAGATAGG 1976

***** ************************************** ** ** ** *****

*AtSTM*  GAGCTTAAAGGACAGCTCTTGCGCAAGTACAGTGGTTACTTAGGGAGCCTCAAGCAAGAG 1184

*BnaA09.STM*  GAGCTTAAAGGACAGCTCTTGCGCAAGTACAGTGGTTACTTAGGCAGTCTGAAGCAAGAG 2034

*BnaC09.STM*  GAGCTTAAAGGACAGCTCTTGCGCAAGTACAGTGGTTACTTAGGCAGTCTGAAGCAAGAG 2036

******************************************** ** ** *********

*AtSTM*  TTCATGAAGAAGAGGAAGAAAGGAAAGCTCCCTAAAGAAGCTCGTCAACAACTGCTTGAT 1244

*BnaA09.STM*  TTCATGAAGAAGAGGAAGAAAGGAAAGCTTCCTAAGGAAGCTCGCCAGCAACTACTTGAC 2094

*BnaC09.STM*  TTCATGAAGAAGAGGAAGAAAGGAAAGCTTCCTAAAGAAGCTCGCCAGCAACTACTTGAC 2096

***************************** ***** ******** ** ***** *****

*AtSTM*  TGGTGGAGCCGTCACTACAAATGGCCTTACCCTTCGGAGCA------------------- 1285

*BnaA09.STM*  TGGTGGAGCCGACACTACAAATGGCCTTACCCTTCGGTAAACTCTTTTCTCTTATTGGCT 2154

*BnaC09.STM*  TGGTGGAGCCGACACTACAAATGGCCTTACCCTTCGGTAAACTCTTTTCTCATATTGGCT 2156

*********** ************************* *

*AtSTM*  ------------------------------------------------------------ 1285

*BnaA09.STM*  ATTATGTTCTTAAATGCATTGCATAAATTGCTTTTACATGATCGCAAATCCCTAGTCAAA 2214

*BnaC09.STM*  ATTATGTTCTTAAATGCATTGCATAAATTGCTTTTACATGATCGCAAATGCCTAGTCAAA 2216

*AtSTM*  ------------------------------------------------------------ 1285

*BnaA09.STM*  GACGAATCAATTGAACATTTTGAGTCAATTTATGTTGTTTTTGTTTATCGGCTCATGCTT 2274

*BnaC09.STM*  GACGAATCAGTTGAACTTTTTGAGTCAATTTATGTTGTTTTTGTCTATCGGCTCATGCTT 2276

*AtSTM*  ------------------------------------------------------------ 1285

*BnaA09.STM*  CCGATGATTCTTGCGTTGCATAAATTGCATTTGCATAATTAGAAATACTAACCAAGATGA 2334

*BnaC09.STM*  CCGATGATTCTTGCGTTGCATAAATTGCATTTGCATAATTAGAAATACTAACCAAGATGA 2336

*AtSTM*  ------------------------------------------------------------ 1285

*BnaA09.STM*  ATGAATTGAACTCATTAATGTAGTCATC----------------CATTCTTACTTCCATA 2378

*BnaC09.STM*  ATGAATTGAACTCATTAATGTAGTCATTCATTAATGTAGTCATCCACTCTTACTTTCATA 2396

*AtSTM*  ------------------------------------------------------------ 1285

*BnaA09.STM*  CGAAATGGTCCGAATAACATACACGTCTTATGGCCA------------------------ 2414

*BnaC09.STM*  CTAAATGGTCCGAATAACATACACGTCTTATGGCCATACTTTAACTTAGCATAAACGTAG 2456

*AtSTM*  ------------------------------------------------------------ 1285

*BnaA09.STM*  -TACTCGTACATCAAGCAATCTCATTGTCGAGTAGTGTGCCTGAAATGCAACTCCCAGTT 2473

*BnaC09.STM*  ATTCTCGTACATCAAGCAATCTCATTGTCGAGTAGTGTGCCTTAAATGCAACTCCCCATT 2516

*AtSTM*  ------------------------------------------------------------ 1285

*BnaA09.STM*  GGCCCCAATGGAATAGTGGCTTAATACATATATATGGTTGATCCTCGTTTGGTATATTTT 2533

*BnaC09.STM*  GGCCCCAATGGAATAGTGGCTTAATACATATATATGGTTGATCCTCGTTTGGTATATTTT 2576

*AtSTM*  ------------------------------------------------------------ 1285

*BnaA09.STM*  CGAATGATCTAATTTAGAAATCCAAATTTATATAGCAACGTTACTTATGCTTTGCACTGA 2593

*BnaC09.STM*  CGAATGATCTAATTTAGAAATCCAAATTTATATAGCAACGTTACTTATGCTTTGCACTGA 2636

*AtSTM*  ------------------------------------------------------------ 1285

*BnaA09.STM*  TTATTCTCAAATAGTTGATGCATCAGAACATATACAACATTGAGAATGTTGATAAGAAAA 2653

*BnaC09.STM*  TTATTCTCAAATAGTTGATGCATCAGAACATATAAAACATTGAGAATGTTGATAAGAAAA 2696

*AtSTM*  --------------------------------------------ACAAAAGCTCGCCCTT 1301

*BnaA09.STM*  CTTAATGATTGATGCCAACTTTTGGATATGAAATGGCAGGAGCAGCAAAAGCTAGCACTA 2713

*BnaC09.STM*  CTTAATGATTGATGCCAACTTTTGGATATGAAATGGCAGGAGCAGCAAAAGCTAGCACTA 2756

******** ** **

*AtSTM*  GCGGAATCAACGGGGCTGGACCAGAAACAGATAAACAATTGGTTCATAAACCAGAGGAAA 1361

*BnaA09.STM*  GCGGAATCAACTGGGCTGGACCAGAAACAGATAAACAATTGGTTCATAAACCAGAGGAAA 2773

*BnaC09.STM*  GCGGAATCAACTGGGCTGGACCAGAAACAGATAAACAACTGGTTCATAAACCAGAGGAAA 2816

*********** ************************** *********************

*AtSTM*  CGGCATTGGAAGCCGTCGGAGGACATGCAGTTTGTAGTAATGGACGCAACACATCCTCAC 1421

*BnaA09.STM*  AGGCACTGGAAACCGTCGGAGGATATGCAGTTTGTAGTAATGGACGCAACACATCCTCAC 2833

*BnaC09.STM*  AGGCATTGGAAACCGTCGGAGGATATGCAGTTTGTAGTAATGGACGCAACACATCCTCAC 2876

**** ***** *********** ************************************

*AtSTM*  CATTACTTCATGGATAATGTCTTGGGCAATCCTTTCCCAATGGATCACATCTCCTCCACC 1481

*BnaA09.STM*  CATTACTTTATGGACAATGTCATGGGAAATCCTTTCCCCATTGATCACATCTCCTCGACC 2893

*BnaC09.STM*  CATTACTTTATGGACAATGTCATGGGAAATCCTTTCCCCATTGATCACATCTCCTCGACC 2936

******** ***** ****** **** *********** ** ************** ***

*AtSTM*  ATGCTTTGATCGATCCTTTAAAACGTTTATTTCATT---ATATTTATAATCGTT-AAACA 1537

*BnaA09.STM*  ATGCTTTGAT---CGATCCTTTGGAAAAACTCTATTTCATAATATTTAATCATT-AAAAG 2949

*BnaC09.STM*  ATGCTTTGATATTCGATCCTTTGGAAAAACTCTATTTCATAATATCTAATCATTAAAAAG 2996

********** * * * *** ** * ***** ** ***

*AtSTM*  GCTTATAATATGTATAATCTATGGTTCTCGGTATACTTGGAACATATGAAACATGTTCCG 1597

*BnaA09.STM*  CTTTGCAATATGTATAATGTATGGTTTCTTGGA--ATACTTAGGAACGTTGCACGTTCCG 3007

*BnaC09.STM*  CTTTGCAATATGTATAATGTATGGTTTCTTGGA--ATACTTAGGAACGTTGCACGTTCCG 3054

** ************ ******* * * * * * * ** ******

*AtSTM*  ATTGATGAACAAGCGTTTGATGTTGTGGTGTGTATCCTTTTGTGTTGTTCTATTGTTATG 1657

*BnaA09.STM*  ATTGATGAACAAGCTTTTGATGTATCCCTTAATTATATATGGGTTGTGTTGTGTGACCTT 3067

*BnaC09.STM*  ATTGATGAACAAGCTTTTGATGTATCCTTTAATTATATATGGGTTGTGTTGTGTGACCTT 3114

************** ******** * * * * * * * ** *

*AtSTM*  CGAAATTCCTATTC--CTATTATAATGATAGTAAAGCTTTTTTGATCTA----------- 1704

*BnaA09.STM*  TTATATTGTTGTTTGTGACTAATGATCTCAACAATGACGCATAAATCTAGAGGGGTGCAT 3127

*BnaC09.STM*  TTGTATTGTTGTGACTAAT----GATCTCAACAATGACGCAACGCATAACTCTAGAGGGT 3170

*** * * ** * ** * *

*AtSTM*  - 1704

*BnaA09.STM*  A 3128

*BnaC09.STM*  G 3171

**Fig. S1** Genomic sequence alignment of two functional copies of *BnaSTM* in J9707 and *STM* in *Arabidopsis thaliana*. The target sequences are underlined with the PAM highlighted in red. "ATG" is marked with yellow background, and "TGA" is marked with green background. "*" indicates positions which have a single, fully conserved residue, "-" are used to indicate the positions of gaps.

*AtSTM*  MESGSNSTSCPMAFAGDNSDGPMCPMMMMMPPIMTSHQ-HHGHDHQHQ-QQEHDGYAYQS 58

*BnaA09.STM* MESGSNSTSCPMAFAGDNSDGPMCPMMMMMMPVITSHQQHHGHDQQHQHQQQHDGYAYQS 60

*BnaC09.STM* MESGSNSTSCPMAFAGDNSDGPMCPMMMMMMPVITSHQQHHGHDQQHQHQQQHDGYAYQS 60

Consensus ****************************** *::**** *****:*** **:********

*AtSTM*  HHQQSSSLFLQSLAPPQ-GTKNKVASSSSPSSCAPAYSLMEIHH-NEIVAGGINPCSSSS 116

*BnaA09.STM* HHQQSSSLFLQSLTPPSQEAKNKVTSSCSPSSGAPAYSFMEINHQNELLAGGLNPCSS-- 118

*BnaC09.STM* HHQHSSLLFLQSLTPPSQEAKNKVRSSCSPSSGAPAYSFMEINHQNELLAGGLNPCSS-- 118

Consensus ***:** ******:**. :**** **.**** *****:***:* **::***:*****

KNOX1

*AtSTM*  SSASVKAKIMAHPHYHRLLAAYVNCQKVGAPPEVVARLEEACSSAAAAAASMGPTGCLGE 176

*BnaA09.STM*  --ASVKAKIMGHPHYHRLLLAYVNCQKVGAPPEVQARLEETCSSAAAAAASMGPTGSLGE 176

*BnaC09.STM*  --ASVKAKIMGHPHYHRLLLAYVNCQKVGAPPEVQARLEETCSSAVAAAASMGPTGSLGE 176

Consensus ********.******** ************** *****:***************.***

KNOX2

*AtSTM*  DPGLDQFMEAYCEMLVKYEQELSKPFKEAMVFLQRVECQFKSLSLSSPSSFS-GYGETAI 235

*BnaA09.STM* DPGLDQFMEAYCEMLVKYEQELSKPFKEAMVFLQHVECQFKSLSLSSPSSFSVGYGEAAI 236

*BnaC09.STM* DPGLDQFMEAYCEMLVKYEQELSKPFKEAMVFLQHVECQFKSLSLSSPSSFSVGYGEAAI 236

Consensus **********************************:***************** ****:**

ELK

*AtSTM*  DRNNNGSSEEEVDMNNEFVDPQAEDRELKGQLLRKYSGYLGSLKQEFMKKRKKGKLPKEA 295

*BnaA09.STM*  ERNNNGSSEEEVDMNNEFVDPQAEDRELKGQLLRKYSGYLGSLKQEFMKKRKKGKLPKEA 296

*BnaC09.STM*  ERNNNGSSEEEVDMNNEFVDPQAEDRELKGQLLRKYSGYLGSLKQEFMKKRKKGKLPKEA 296

Consensus :***********************************************************

HD

*AtSTM*  RQQLLDWWSRHYKWPYPSEQQKLALAESTGLDQKQINNWFINQRKRHWKPSEDMQFVVMD 355

*BnaA09.STM*  RQQLLDWWSRHYKWPYPSEQQKLALAESTGLDQKQINNWFINQRKRHWKPSEDMQFVVMD 356

*BnaC09.STM*  RQQLLDWWSRHYKWPYPSEQQKLALAESTGLDQKQINNWFINQRKRHWKPSEDMQFVVMD 356

Consensus ************************************************************

*AtSTM*  ATHPHHYFMDNVLGNPFPMDHISSTML 382

*BnaA09.STM*  ATHPHHYFMDNVMGNPFPIDHISSTML 383

*BnaC09.STM*  ATHPHHYFMDNVMGNPFPIDHISSTML 383

Consensus ************:*****:********

**Fig. S2 Alignment of *STM* homolog protein** **sequences identified from *B. napus* (*BnaA09.STM, BnaC09.STM*) and** ***A. thaliana* (*AtSTM*).** The *KNOX1* conserved domain is highlighted in light gray, the KNOX2 conserved domain is highlighted in dark gray, the ELK conserved domain is highlighted in cyan, and the conserved Homeodomain is highlighted in yellow color. Red fonts show differences in amino acids. "*" indicates positions which have a single, fully conserved residue, ":" indicates that one of the following 'strong' groups is fully conserved.


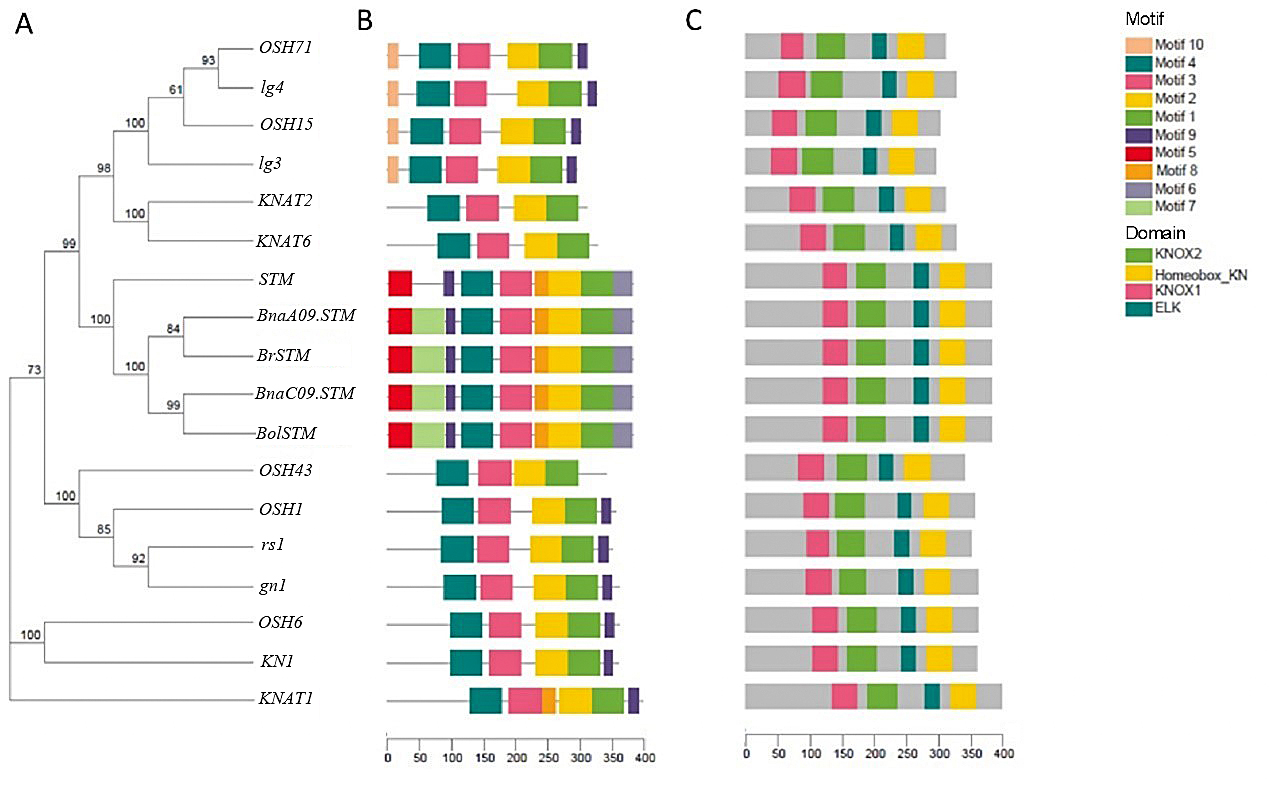


**Fig. S3** **Phylogenetic tree of the *KNOX Ⅰ* gene family in main crops.** A: The phylogenetic tree is generated from the alignment of the whole protein sequences after 1000 bootstrap repeats. B: The motif prediction results, and the motifs are distinguished by different display colors. C: The prediction result of the conserved domain, and the domain are distinguished by different display colors (Green: KNOX2, yellow: Homeobox_KN, red: KNOX1, teal: ELK). Among them, *KNAT1*, *KNAT2*, *KNAT6*, *STM* are derived from *Arabidopsis thaliana*, *BnaA09.STM* and *BnaC09.STM* is derived from *B. napus*, *BrSTM* and *BolSTM* is derived from *B. rapa* and *B. oleracea*, respectively. *OSH1*, *OSH6*, *OSH15*, *OSH43*, *OSH71* are derived from *Oryza sativa*. *KN1*, *rs1*, *gn1*, *lg3*, and *lg4* are derived from *Zea mays*.


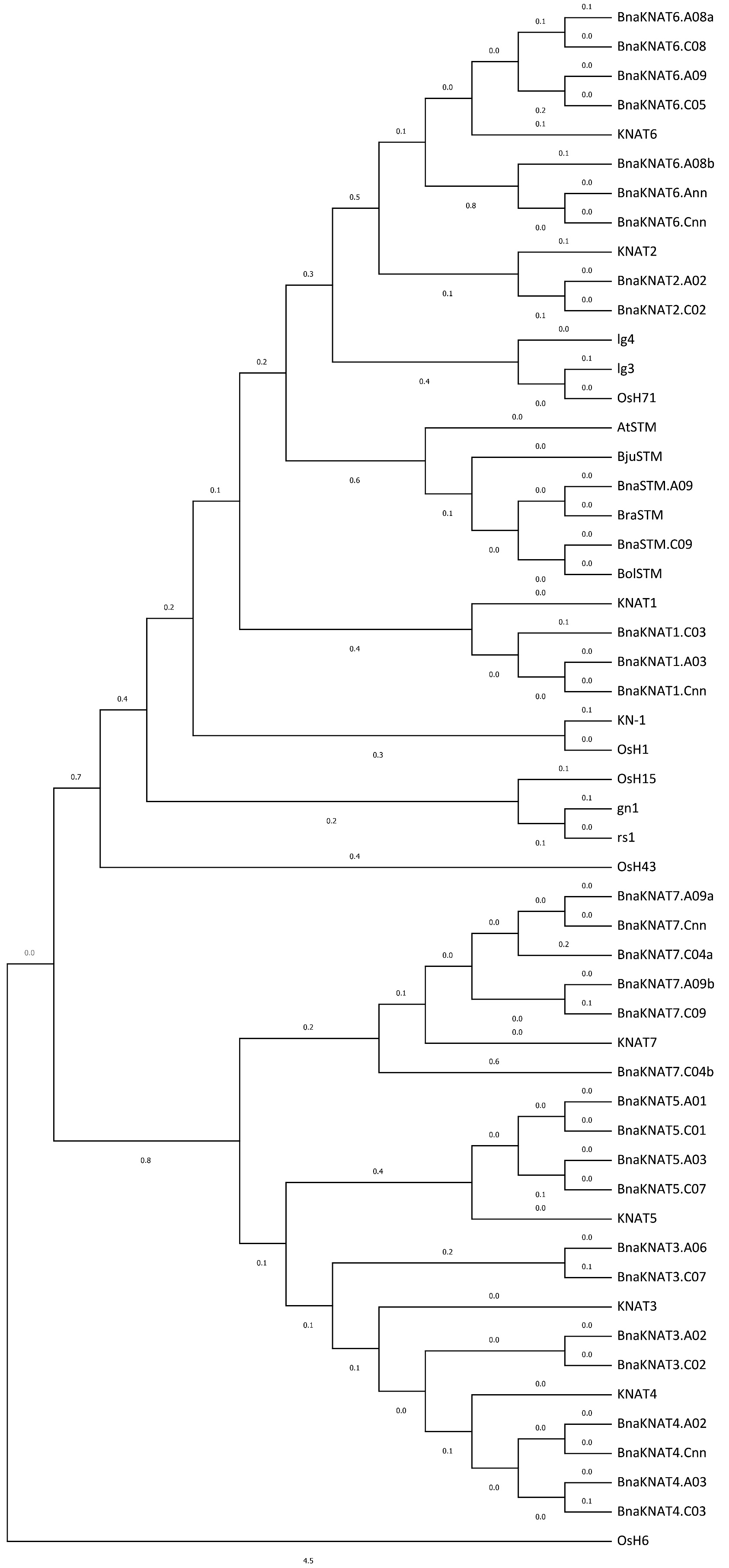


**Fig. S4 Phylogenetic tree showing the sequence relationship among *STM* homologs identified from various plant species.** The protein sequences were obtained from the GenBank with the following accession numbers: *AtSTM* (Q38874), *KNAT1* (P46639), *KNAT2* (P46640), *KNAT3* (P48000), *KNAT4* (P48001), *KNAT5* (P48002), *KNAT6* (Q84JS6), *KNAT7* (Q9FPQ8) in *Arabidopsis thaliana*; *BnaC03.KNAT1* (CDY34012), *BnaCnn.KNAT1* (CDY68624), *BnaA03.KNAT1* (KAH0933309), *BnaC03.KNAT2* (CDY09448), *BnaA02.KNAT2* (KAH0938085), *BnaC02.KNAT3* (KAH0899362), *BnaA02.KNAT3* (KAH0939801), *BnaC07.KNAT3* (KAH0869679), *BnaA06.KNAT3* (KAH0923281), *BnaCnn.KNAT4* (CDY51104), *BnaA03.KNAT4* (KAH0931268), *BnaC03.KNAT4* (KAH0888026), *BnaA02.KNAT4* (KAH0936672), *BnaA03.KNAT5* (CDY13472), *BnaC07.KNAT5* (KAH0871091), *BnaA01.KNAT5* (KAH0940850), *BnaC01.KNAT5* (KAH0901053), *BnaA08a.KNAT6* (CDY41448), *BnaA08b.KNAT6* (CDY41449), *BnaA09.KNAT6* (CDY44142), *BnaAnn.KNAT6* (CDY69524), *BnaC05.KNAT6* (KAH0878209), *BnaC08.KNAT6* (KAH0862404), *BnaCnn.KNAT6* (CDY70923), *BnaA09a.KNAT7* (CDY64892), *BnaCnn.KNAT7* (CDY66559), *BnaC09.KNAT7* (CDY45472), *BnaA09b.KNAT7* (CDY45968), *BnaC04a.KNAT7* (KAH0883752), *BnaC04b.KNAT7* (KAH0883754), *BnaA08.STM* (CDY12943) and *BnaC09.STM* (CDY19545) in *Brassica napus*; *BolSTM* ([AAM89270.1](https://www.ncbi.nlm.nih.gov/protein/AAM89270.1?report=genbank&log$=prottop&blast_rank=12&RID=HY2VTPWU016)) in *Brassica oleracea*; *BjuSTM* (P[454609](https://www.uniprot.org/uniprot/P46609)) in *Brassica juncea*; *BrSTM* ([RID44679.1](https://www.ncbi.nlm.nih.gov/protein/RID44679.1?report=genbank&log$=prottop&blast_rank=1&RID=HY2VTPWU016)) in *Brassica rapa*; *OsH1* (P[46609](https://www.uniprot.org/uniprot/P46609)), *OsH6* ([Q02201](https://www.uniprot.org/uniprot/Q02201)), *OsH15* (O80416), *OsH71* (Q7GDL5), *OsH43* (Q10ED2) in *Oryza sativa subsp.* *japonica*; *rs1* (Q41853), *gn1* (Q7XYR8), *KN-1* (P24345), *lg3* (P56669), *lg4* (P56668) in *Zea mays*.

**A**

**STM-8-5-1-9 aacc T3**

>aa

*BnaA09.STM* MESGSNSTSCPMAFAGDNSDGPMCPMMMMMMPVITSHQQHHGHDQQHQHQQQHDGYAYQS 60

aa MESGSNSTSCPMAFAGDNSDGPMCPMMMMMMPVITSHQQHHGHDQQHQHQQQHDGYAYQS 60

*BnaA09.STM* HHQQSSSLFLQSLTPPSQEAKNKVTSSCSPSSGAPAYSFMEINHQNELLAGGLNPCSSAS 120

aa HHQQSSSLFLQSLTPPSQEAKNKVTSSCSPSFWCSCLFFHGDQ----------SPKRT-- 108

*BnaA09.STM* VKAKIMGHPHYHRLLLAYVNCQKVGA-PPEVQARLEETCSSAAAAAASMGPTGSLG---- 175

aa --------PRRRTQSLFFSLCQGQNHGSSSLPPPLTRLCQLPEG-GSSTGSAGEAGRNML 159

*BnaA09.STM* ------EDPGLDQFMEAYCEMLVKYEQELSKPFKEAMVFLQHVECQFKSLSLSSPSSFSV 229

aa VCGGSRSVDGTHRFFR* 175

*BnaA09.STM* GYGEAAIERNNNGSSEEEVDMNNEFVDPQAEDRELKGQLLRKYSGYLGSLKQEFMKKRKK 289

*BnaA09.STM* GKLPKEARQQLLDWWSRHYKWPYPSEQQKLALAESTGLDQKQINNWFINQRKRHWKPSED 349

*BnaA09.STM* MQFVVMDATHPHHYFMDNVMGNPFPIDHISSTML 383

>cc

*BnaC09.STM* MESGSNSTSCPMAFAGDNSDGPMCPMMMMMMPVITSHQQHHGHDQQHQHQQQHDGYAYQS 60

cc MESGSNSTSCPMAFAGDNSDGPMCPMMMMMMPVITSHQQHHGHDQQHQHQQQHDGYAYQS 60

*BnaC09.STM* HHQHSSLLFLQSLTPPSQEAKNKVRSSCSPSSGAPAYSFMEINHQNELLAGGLNPCSSAS 120

cc HHQHSSLLFLQSLTPPSQEAKNKVRSSCSPSIWCSCLFFHGDQ----------SPKRT-- 108

*BnaC09.STM* VKAKIMGHPHYHRLLLAYVNCQKVGAP-PEVQARLEETCSSAAAAAASMGPTGSLGEDPG 179

cc --------PRRRTQSLFFSLCQGQNHGSSSLPPPLARLCQLPE-GGSSTGSAGEAGRN-- 157

*BnaC09.STM* LDQFMEAYCEMLVKYEQELSKPFKEAMVFLQHVECQFKSLSLSSPSSFSVGYGEAAIERN 239

cc ---MLVCGCRRSVDGTHRF---FR*----------------------------------- 175

*BnaC09.STM* NNGSSEEEVDMNNEFVDPQAEDRELKGQLLRKYSGYLGSLKQEFMKKRKKGKLPKEARQQ 299

*BnaC09.STM* LLDWWSRHYKWPYPSEQQKLALAESTGLDQKQINNWFINQRKRHWKPSEDMQFVVMDATH 359

*BnaC09.STM* PHHYFMDNVMGNPFPIDHISSTML 383

**B**

**STM-28-4-2-7 aacc T3**

>aa

*BnaA09.STM* MESGSNSTSCPMAFAGDNSDGPMCPMMMMMMPVITSHQQHHGHDQQHQHQQQHDGYAYQS 60

aa MESGSNSTSCSNGFCRG* 17

*BnaA09.STM* HHQQSSSLFLQSLTPPSQEAKNKVTSSCSPSSGAPAYSFMEINHQNELLAGGLNPCSSAS 120

*BnaA09.STM* VKAKIMGHPHYHRLLLAYVNCQKVGAPPEVQARLEETCSSAAAAAASMGPTGSLGEDPGL 180

*BnaA09.STM* DQFMEAYCEMLVKYEQELSKPFKEAMVFLQHVECQFKSLSLSSPSSFSVGYGEAAIERNN 240

*BnaA09.STM* NGSSEEEVDMNNEFVDPQAEDRELKGQLLRKYSGYLGSLKQEFMKKRKKGKLPKEARQQL 300

*BnaA09.STM* LDWWSRHYKWPYPSEQQKLALAESTGLDQKQINNWFINQRKRHWKPSEDMQFVVMDATHP 360

*BnaA09.STM* HHYFMDNVMGNPFPIDHISSTML 383

>cc

*BnaC09.STM* MESGSNSTSCPMAFAGDNSDGPMCPMMMMMMPVITSHQQHHGHDQQHQHQQQHDGYAYQS 60

cc MESGSNSTSCPMAFAGDNSDGPMCPMMMMMMPVITSHQQHHGHDQQHQHQQQHDGYAYQS 60

*BnaC09.STM* HHQHSSLLFLQSLTPPSQEAKNKVRSSCSPSSGAPAYSFMEINHQNELLAGGLNPCSSAS 120

cc HHQHSSLLFLQSLTPPSQEAKNKVRSSCSPLVLLLILSWRSITKTNSSQEDSIPVL---- 116

*BnaC09.STM* VKAKIMGHPHYHRLLLAYVNCQKVGAPPEVQARLEETCSSAAAAAASMGPTGSLGEDPGL 180

cc ---QPLSRPKSWVILTTTASCSPMSIARR--WELHRKCRRGWKKHARLRLPPQRRWDPQV 171

*BnaC09.STM* DQFMEAYCEMLVKYEQELSKPFKEAMVFLQHVECQFKSLSLSSPSSFSVGYGEAAIERNN 240

cc L* 172

*BnaC09.STM* NGSSEEEVDMNNEFVDPQAEDRELKGQLLRKYSGYLGSLKQEFMKKRKKGKLPKEARQQL 300

*BnaC09.STM* LDWWSRHYKWPYPSEQQKLALAESTGLDQKQINNWFINQRKRHWKPSEDMQFVVMDATHP 360

*BnaC09.STM* HHYFMDNVMGNPFPIDHISSTML 383

**C**

**STM-11-7-8-14 aacc T3**

>aa

*BnaA09.STM* MESGSNSTSCPMAFAGDNSDGPMCPMMMMMMPVITSHQQHHGHDQQHQHQQQHDGYAYQS 60

aa MESGSNSTSCLNGFCRG* 17

*BnaA09.STM* HHQQSSSLFLQSLTPPSQEAKNKVTSSCSPSSGAPAYSFMEINHQNELLAGGLNPCSSAS 120

*BnaA09.STM* VKAKIMGHPHYHRLLLAYVNCQKVGAPPEVQARLEETCSSAAAAAASMGPTGSLGEDPGL 180

*BnaA09.STM* DQFMEAYCEMLVKYEQELSKPFKEAMVFLQHVECQFKSLSLSSPSSFSVGYGEAAIERNN 240

*BnaA09.STM* NGSSEEEVDMNNEFVDPQAEDRELKGQLLRKYSGYLGSLKQEFMKKRKKGKLPKEARQQL 300

*BnaA09.STM* LDWWSRHYKWPYPSEQQKLALAESTGLDQKQINNWFINQRKRHWKPSEDMQFVVMDATHP 360

*BnaA09.STM* HHYFMDNVMGNPFPIDHISSTML 383

>cc

*BnaC09.STM* MESGSNSTSCPMAFAGDNSDGPMCPMMMMMMPVITSHQQHHGHDQQHQHQQQHDGYAYQS 60

cc MESGSNSTSCLNGFCRG* 17

*BnaC09.STM* HHQHSSLLFLQSLTPPSQEAKNKVRSSCSPSSGAPAYSFMEINHQNELLAGGLNPCSSAS 120

*BnaC09.STM* VKAKIMGHPHYHRLLLAYVNCQKVGAPPEVQARLEETCSSAAAAAASMGPTGSLGEDPGL 180

*BnaC09.STM* DQFMEAYCEMLVKYEQELSKPFKEAMVFLQHVECQFKSLSLSSPSSFSVGYGEAAIERNN 240

*BnaC09.STM* NGSSEEEVDMNNEFVDPQAEDRELKGQLLRKYSGYLGSLKQEFMKKRKKGKLPKEARQQL 300

*BnaC09.STM* LDWWSRHYKWPYPSEQQKLALAESTGLDQKQINNWFINQRKRHWKPSEDMQFVVMDATHP 360

*BnaC09.STM* HHYFMDNVMGNPFPIDHISSTML 383

**D**

**STM-14-1-11-9 aacc T3**

>aa

*BnaA09.STM* MESGSNSTSCPMAFAGDNSDGPMCPMMMMMMPVITSHQQHHGHDQQHQHQQQHDGYAYQS 60

aa MESGSNSTSCLNGFCRG* 17

*BnaA09.STM* HHQQSSSLFLQSLTPPSQEAKNKVTSSCSPSSGAPAYSFMEINHQNELLAGGLNPCSSAS 120

*BnaA09.STM* VKAKIMGHPHYHRLLLAYVNCQKVGAPPEVQARLEETCSSAAAAAASMGPTGSLGEDPGL 180

*BnaA09.STM* DQFMEAYCEMLVKYEQELSKPFKEAMVFLQHVECQFKSLSLSSPSSFSVGYGEAAIERNN 240

*BnaA09.STM* NGSSEEEVDMNNEFVDPQAEDRELKGQLLRKYSGYLGSLKQEFMKKRKKGKLPKEARQQL 300

*BnaA09.STM* LDWWSRHYKWPYPSEQQKLALAESTGLDQKQINNWFINQRKRHWKPSEDMQFVVMDATHP 360

*BnaA09.STM* HHYFMDNVMGNPFPIDHISSTML 383

>cc

*BnaC09.STM* MESGSNSTSCPMAFAGDNSDGPMCPMMMMMMPVITSHQQHHGHDQQHQHQQQHDGYAYQS 60

cc MESGSNSTSCLNGFCRG* 17

*BnaC09.STM* HHQHSSLLFLQSLTPPSQEAKNKVRSSCSPSSGAPAYSFMEINHQNELLAGGLNPCSSAS 120

*BnaC09.STM* VKAKIMGHPHYHRLLLAYVNCQKVGAPPEVQARLEETCSSAAAAAASMGPTGSLGEDPGL 180

*BnaC09.STM* DQFMEAYCEMLVKYEQELSKPFKEAMVFLQHVECQFKSLSLSSPSSFSVGYGEAAIERNN 240

*BnaC09.STM* NGSSEEEVDMNNEFVDPQAEDRELKGQLLRKYSGYLGSLKQEFMKKRKKGKLPKEARQQL 300

*BnaC09.STM* LDWWSRHYKWPYPSEQQKLALAESTGLDQKQINNWFINQRKRHWKPSEDMQFVVMDATHP 360

*BnaC09.STM* HHYFMDNVMGNPFPIDHISSTML 383

**E**

**STM-8-5-10-21 aaCC T3**

>aa

*BnaA09.STM* MESGSNSTSCPMAFAGDNSDGPMCPMMMMMMPVITSHQQHHGHDQQHQHQQQHDGYAYQS 60

aa MESGSNSTSCLNGFCRG* 17

*BnaA09.STM* HHQQSSSLFLQSLTPPSQEAKNKVTSSCSPSSGAPAYSFMEINHQNELLAGGLNPCSSAS 120

*BnaA09.STM* VKAKIMGHPHYHRLLLAYVNCQKVGAPPEVQARLEETCSSAAAAAASMGPTGSLGEDPGL 180

*BnaA09.STM* DQFMEAYCEMLVKYEQELSKPFKEAMVFLQHVECQFKSLSLSSPSSFSVGYGEAAIERNN 240

*BnaA09.STM* NGSSEEEVDMNNEFVDPQAEDRELKGQLLRKYSGYLGSLKQEFMKKRKKGKLPKEARQQL 300

*BnaA09.STM* LDWWSRHYKWPYPSEQQKLALAESTGLDQKQINNWFINQRKRHWKPSEDMQFVVMDATHP 360

*BnaA09.STM* HHYFMDNVMGNPFPIDHISSTML 383

**F**

**STM-14-8-12-5 aaCC T3**

>aa

*BnaA09.STM* MESGSNSTSCPMAFAGDNSDGPMCPMMMMMMPVITSHQQHHGHDQQHQHQQQHDGYAYQS 60

aa MESGSNSTSCLNGFCRG* 17

*BnaA09.STM* HHQQSSSLFLQSLTPPSQEAKNKVTSSCSPSSGAPAYSFMEINHQNELLAGGLNPCSSAS 120

*BnaA09.STM* VKAKIMGHPHYHRLLLAYVNCQKVGAPPEVQARLEETCSSAAAAAASMGPTGSLGEDPGL 180

*BnaA09.STM* DQFMEAYCEMLVKYEQELSKPFKEAMVFLQHVECQFKSLSLSSPSSFSVGYGEAAIERNN 240

*BnaA09.STM* NGSSEEEVDMNNEFVDPQAEDRELKGQLLRKYSGYLGSLKQEFMKKRKKGKLPKEARQQL 300

*BnaA09.STM* LDWWSRHYKWPYPSEQQKLALAESTGLDQKQINNWFINQRKRHWKPSEDMQFVVMDATHP 360

*BnaA09.STM* HHYFMDNVMGNPFPIDHISSTML 383

**G**

**STM-11-10-3-6 AAcc T3**

>cc

*BnaC09.STM* MESGSNSTSCPMAFAGDNSDGPMCPMMMMMMPVITSHQQHHGHDQQHQHQQQHDGYAYQS 60

cc MESGSNSTSCPMAFAGDNSDGPMCPMMMMMMPVITSHQQHHGHDQQHQHQQQHDGYAYQS 60

*BnaC09.STM* HHQHSSLLFLQSLTPPSQEAKNKVRSSCSPSSGAPAYSFMEINHQNELLAGGLNPCSSAS 120

cc HHQHSSLLFLQSLTPPSQEAKNKVRSSCSPLVLLLILSWRSITKTNSSQEDSIPVL---- 116

*BnaC09.STM* VKAKIMGHPHYHRLLLAYVNCQKVGAPPEVQARLEETCSSAAAAAASMGPTGSLGEDPGL 180

cc ---QPLSRPKSWVILTTTASCSPMSIARR--WELHRKCRRGWKKHARLRLPPQRRWDPQV 171

*BnaC09.STM* DQFMEAYCEMLVKYEQELSKPFKEAMVFLQHVECQFKSLSLSSPSSFSVGYGEAAIERNN 240

cc L* 172

*BnaC09.STM* NGSSEEEVDMNNEFVDPQAEDRELKGQLLRKYSGYLGSLKQEFMKKRKKGKLPKEARQQL 300

*BnaC09.STM* LDWWSRHYKWPYPSEQQKLALAESTGLDQKQINNWFINQRKRHWKPSEDMQFVVMDATHP 360

*BnaC09.STM* HHYFMDNVMGNPFPIDHISSTML 383

**H**

**STM-7-7-9-2 AAcc T3**

>cc

*BnaC09.STM* MESGSNSTSCPMAFAGDNSDGPMCPMMMMMMPVITSHQQHHGHDQQHQHQQQHDGYAYQS 60

cc MESGSNSTSCPMAFAGDNSDGPMCPMMMMMMPVITSHQQHHGHDQQHQHQQQHDGYAYQS 60

*BnaC09.STM* HHQHSSLLFLQSLTPPSQEAKNKVRSSCSPSSGAPAYSFMEINHQNELLAGGLNPCSSAS 120

cc HHQHSSLLFLQSLTPPSQEAKNKVRSSCSPSIWCSCLFFHGDQ----------SPKRT-- 108

*BnaC09.STM* VKAKIMGHPHYHRLLLAYVNCQKVGAP-PEVQARLEETCSSAAAAAASMGPTGSLGEDPG 179

cc --------PRRRTQSLFFSLCQGQNHGSSSLPPPLARLCQLPE-GGSSTGSAGEAGRN-- 157

*BnaC09.STM* LDQFMEAYCEMLVKYEQELSKPFKEAMVFLQHVECQFKSLSLSSPSSFSVGYGEAAIERN 239

cc ---MLVCGCRRSVDGTHRF---FR* 175

*BnaC09.STM* NNGSSEEEVDMNNEFVDPQAEDRELKGQLLRKYSGYLGSLKQEFMKKRKKGKLPKEARQQ 299

*BnaC09.STM* LLDWWSRHYKWPYPSEQQKLALAESTGLDQKQINNWFINQRKRHWKPSEDMQFVVMDATH 359

*BnaC09.STM* PHHYFMDNVMGNPFPIDHISSTML 383

**Fig. S5** The predicted amino acid sequences of *BnaSTM* homozygous mutants in the T3 generation. STM-8–5–1, STM-28–4–2 (B), STM-11–7–8 (C), STM-14–1–11 (D), are double-homozygous mutant lines of *BnaSTM*; STM-8–5–10–21–6 (E), STM-14–8–12–5 (F), are single-homozygous mutant lines of *BnaA09.STM*; STM-11–10–3–6 (G), STM-7–7–9–2 (H), are single-homozygous mutant lines of *BnaC09.STM*. The stars indicate stop codon, and numbers indicate amino acid positions; red letters indicate frameshift amino acids; “-” indicate deletion of amino acid; “aa” and “cc” represent the homozygous mutated alleles of the target gene on *BnaA09.STM* and *BnaC09.STM*, respectively. The “aaCC”, “AAcc” and “aacc” represent homozygous mutations of the target gene in *BnaA09.STM*, *BnaC09.STM,* and both copies, respectively.


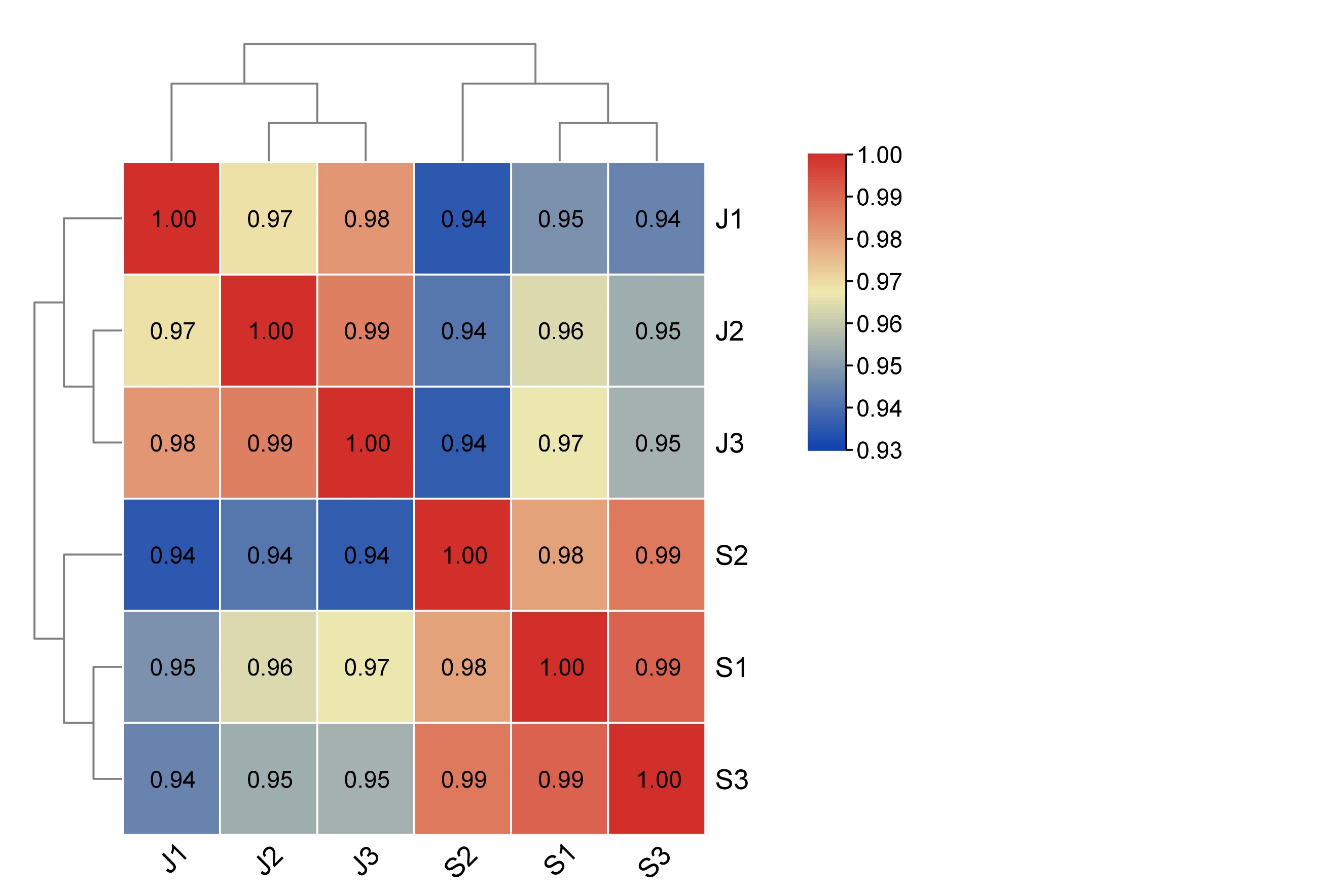


**Fig. S6** Pearson correlation coefficient among counts of transcriptome data.

A


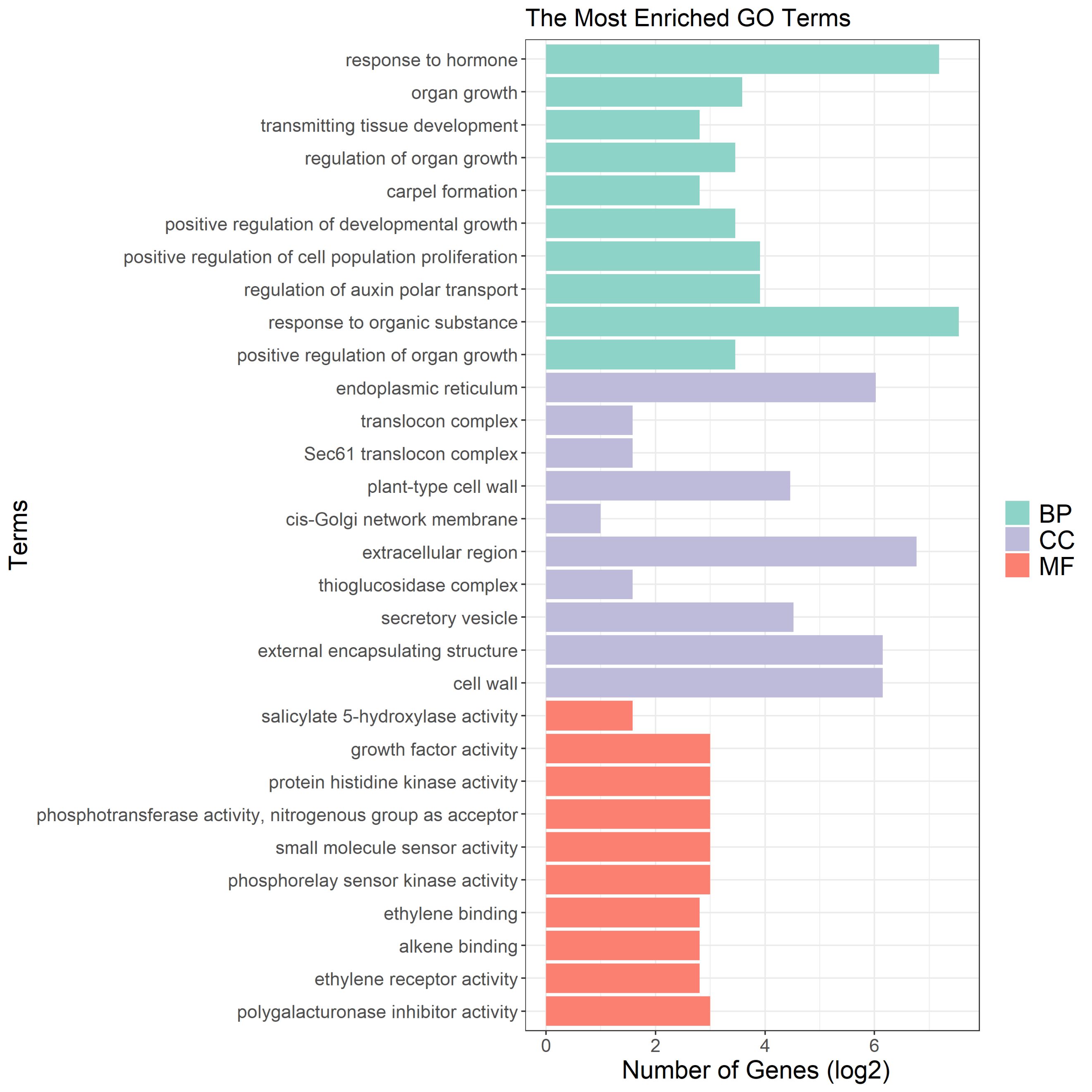


B


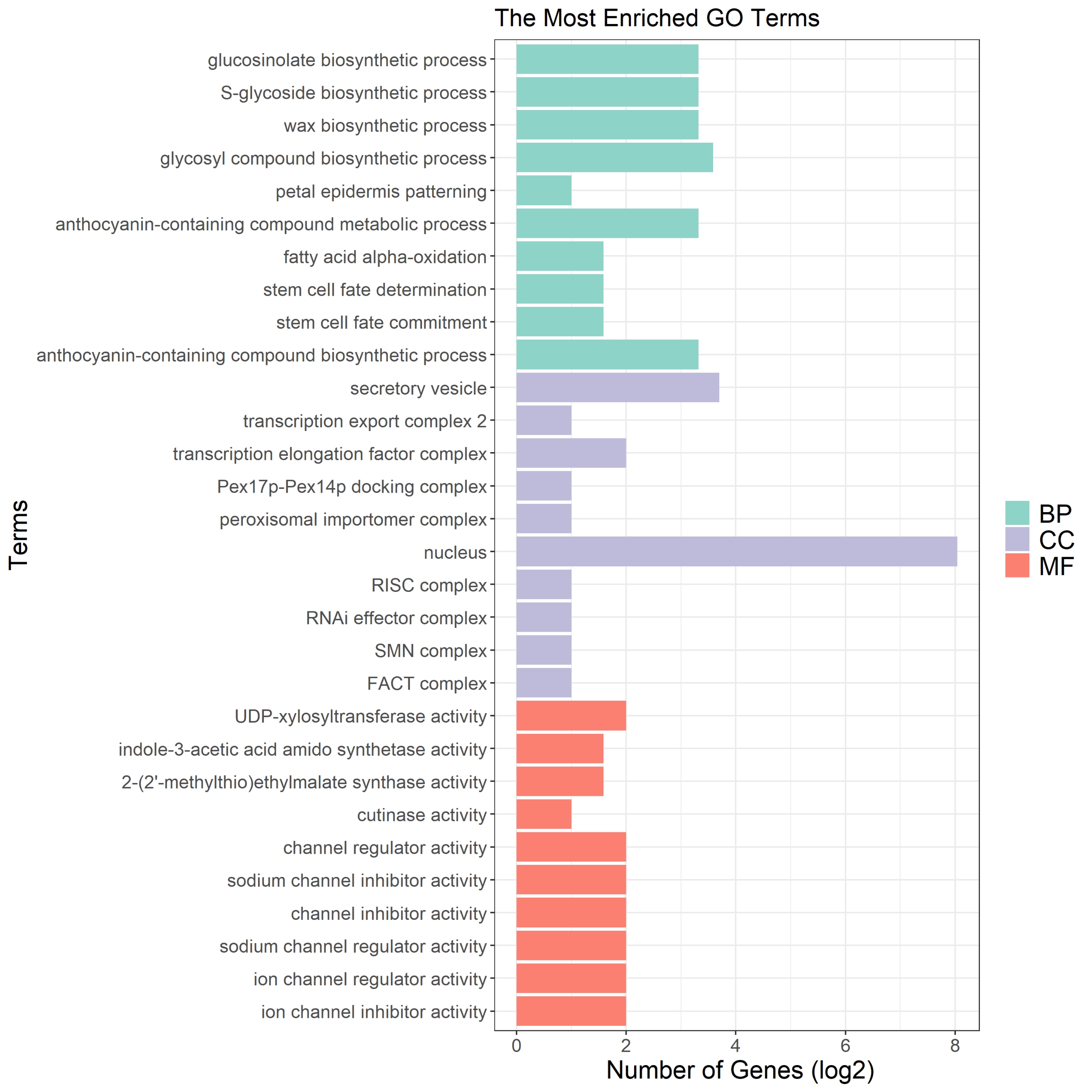


**Fig. S7** Results of GO annotation of all up-and down-regulated genes. (A) GO Annotation Map of up-regulated Genes (B) GO Annotation Map of down-regulated Genes.

A

B

**Fig. S8** Results of Top 20 GO annotation of all up-and down-regulated genes. (A) Top 20 GO Annotation Map of up-DEGs; (B) Top 20 GO Annotation Map of down-DEGs.

A


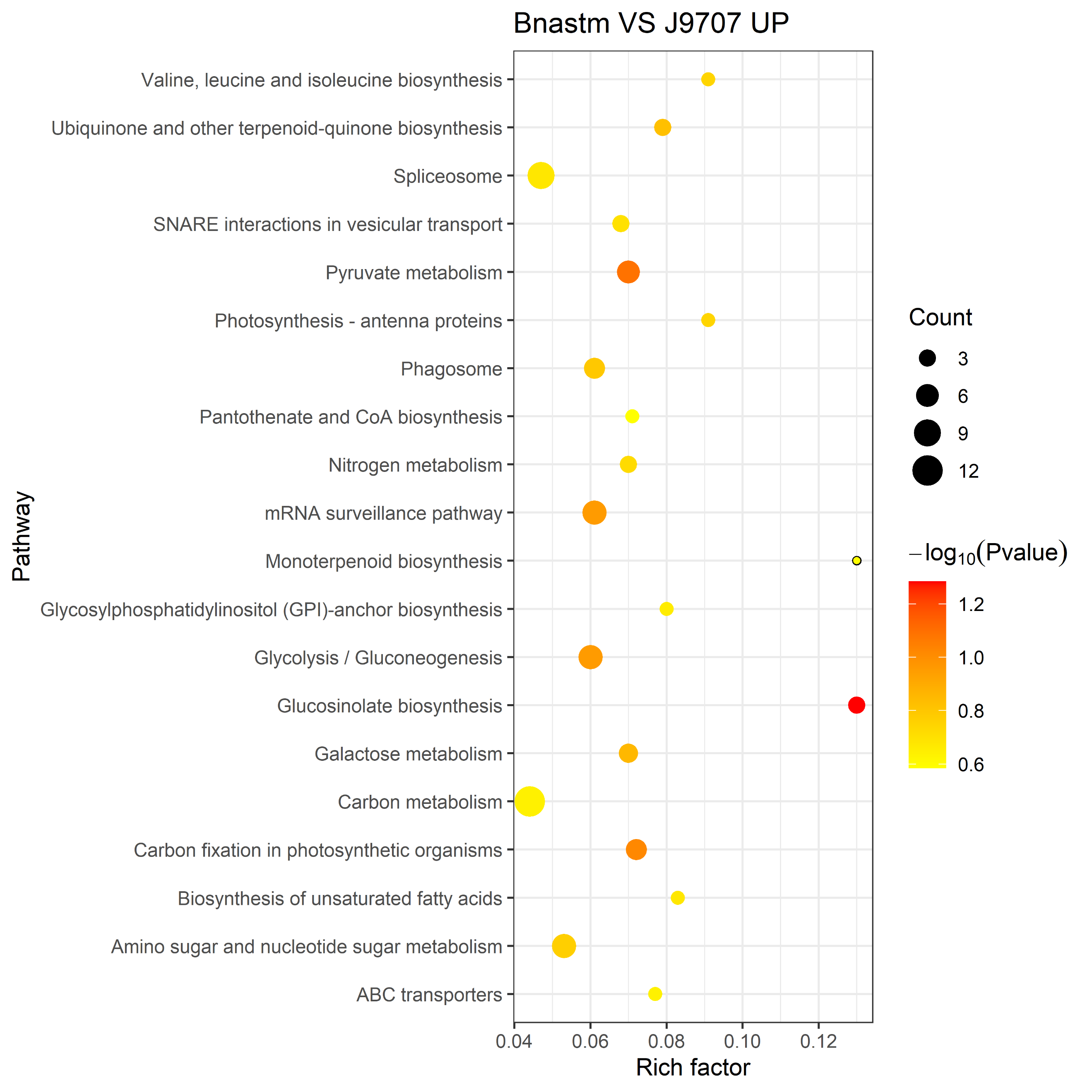


B


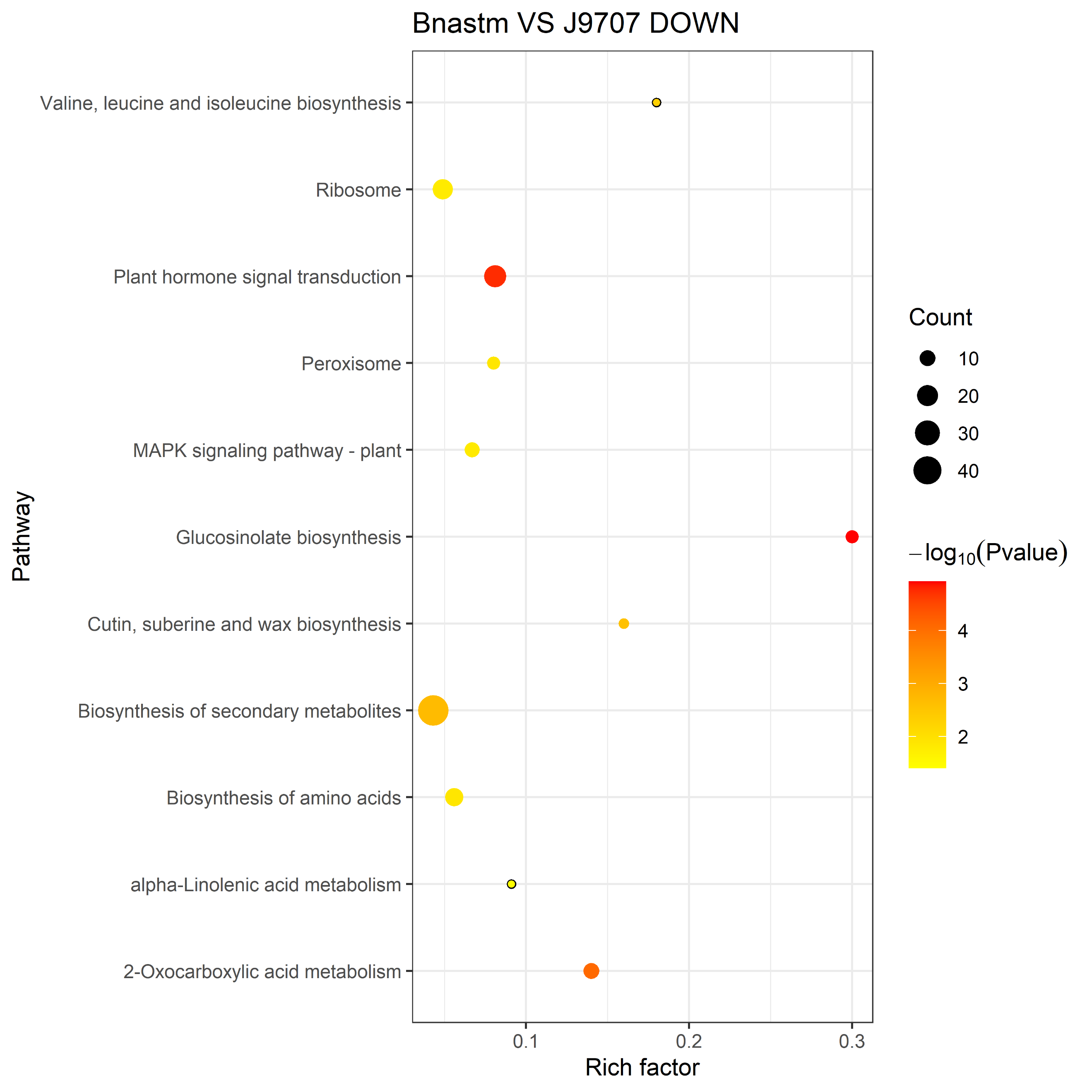


**Fig. S9** Results of KEGG pathway of all up-and down-regulated genes. (A) KEGG pathway of up-DEGs; (B) KEGG pathway of down-DEGs.

**Fig. S10** Correlation of gene expression changes from qPCR and RNA-seq methods at petal. The log2 value of the expression ratio (aacc/WT; y-axis) in the 1D analysis was plotted against the log2 fold change from the RNA-seq data (x-axis), The Internal reference of qPCR is BnUBC9.
